# Long Bone Histology of Sauropterygia from the Lower Muschelkalk of the Germanic Basin Provides Unexpected Implications for Phylogeny

**DOI:** 10.1371/journal.pone.0011613

**Published:** 2010-07-21

**Authors:** Nicole Klein

**Affiliations:** Steinmann Institute of Geology, Mineralogy and Palaeontology, University of Bonn, Bonn, Germany; Raymond M. Alf Museum of Paleontology, United States of America

## Abstract

**Background:**

Sauropterygia is an abundant and successful group of Triassic marine reptiles. Phylogenetic relationships of Triassic Sauropterygia have always been unstable and recently questioned. Although specimens occur in high numbers, the main problems are rareness of diagnostic material from the Germanic Basin and uniformity of postcranial morphology of eosauropterygians. In the current paper, morphotypes of humeri along with their corresponding bone histologies for Lower to Middle Muschelkalk sauropterygians are described and interpreted for the first time in a phylogenetic context.

**Methodology/Principal Findings:**

*Nothosaurus* shows a typical plesiomorphic lamellar-zonal bone type, but varying growth patterns and the occurrence of a new humerus morphotype point to a higher taxonomic diversity than was known. In contrast to the enormous morphological variability of eosauropterygian humeri not assigned to *Nothosaurus*, their long bone histology is relatively uniform and can be divided into two histotypes. Unexpectedly, both of these histotypes reveal abundant fibrolamellar bone throughout the cortex. This pushes the origin of fibrolamellar bone in Sauropterygia back from the Cretaceous to the early Middle Triassic (early Anisian). Histotype A is assigned to *Cymatosaurus*, a basal member of the Pistosauroidea, which includes the plesiosaurs as derived members. Histotype B is related to the pachypleurosaur *Anarosaurus*. Contrary to these new finds, the stratigraphically younger pachypleurosaur *Neusticosaurus* shows the plesiomorphic lamellar-zonal bone type and an incomplete endochondral ossification, like *Nothosaurus*.

**Conclusions/Significance:**

Histological results hypothetically discussed in a phylogenetical context have a large impact on the current phylogenetic hypothesis of Sauropterygia, leaving the pachypleurosaurs polyphyletic. On the basis of histological data, *Neusticosaurus* would be related to *Nothosaurus*, whereas *Anarosaurus* would follow the pistosaur clade. Furthermore, the presence of fibrolamellar bone, which is accompanied with increased growth rates and presumably even with increased metabolic rates, already in *Anarosaurus* and *Cymatosaurus* can explain the success of the Pistosauroidea, the only sauropterygian group to survive into the Jurassic and give rise to the pelagic plesiosaur radiation.

## Introduction

### General Introduction

**Figure 1:**
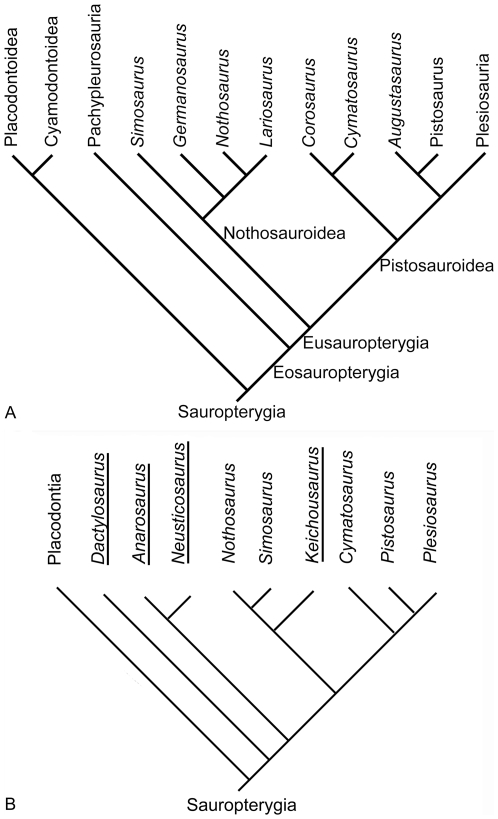
Phylogenetic relationships of Sauropterygia. **1A**) **Current phylogenetic relationships of Sauropterygia.** Phylogenetic hypothesis of early Sauropterygia summarized after Rieppel [1]. Hereafter the Nothosauroidea and Pistosauroidea (incl. *Cymatosaurus*) form the Eusauropterygia, which are combined with the Pachypleurosauroidea in the Eosauropterygia. Placodonts are regarded as a plesiomorphic sister group to all other Sauropterygia. All these groups appear nearly simultaneously in the early Anisian (early Middle Triassic). Nothosaurs and pachypleurosaurs vanish in the Ladinian (late Middle Triassic). Placodonts are known until the Rhaetian (Late Triassic). Among the Pistosauroidea *Cymatosaurus* is restricted to the Anisian, pistosaurs disappear in the Carnian (early Late Triassic). Plesiosauria are known from the Rhaetian (Late Triassic) until the end of the Cretaceous. **1B**) **Sauropterygian relationships modified after Holmes et al.** [**8**] **and own unpublished data.** Holmes et al. suggested that the pachypleurosaur *Keichousaurus* is possibly a basal nothosaurid, questioning the monophyly of Pachypleurosauria [8]. The interrelationships of the Lower Muschelkalk pachypleurosaurs *Dactylosaurus* and *Anarosaurus* are also unclear (own data). Pachypleurosaur taxa are underlined in this figure.

Sauropterygians are a group of extinct, marine reptiles that invaded the sea in the Early Triassic and became extinct in the Late Cretaceous. They are diapsid reptiles and most likely belong to the Lepidosauromorpha [1]. Although Sauropterygia is best known for the large Jurassic and Cretaceous plesiosaurs, the Sauropterygia was much more diverse during the Triassic, containing the Placodontia, the Pachypleurosauria, the Nothosauroidea, and the Pistosauroidea (Fig. 1A), which includes the Jurassic and Cretaceous Plesiosauria (Fig. 1A). Whereas the medium-sized (approximately 0.6 m to 2 m) placodonts were durophagous and feed on hard-shelled invertebrates, the small pachypleurosaurs (approximately 0.25 m to 1.20 m), the medium-sized to large nothosaurs (approximately 1.5 m to 6 m), and the medium-sized pistosaurs (approximately 3 m to 4 m) most likely fed on anything they were able to subdue, such as invertebrates, fishes, and small marine reptiles [2].

Sauropterygians first appeared in the fossil record of the Germanic Basin within the Upper Buntsandstein (late Olenekian, Early Triassic). However, the origin and ancestry of Sauropterygia are largely unknown [1], [3]. After their first appearance, they radiated and diversified successfully through the Tethys and their epicontinental seas. In the Lower Muschelkalk (early Anisian, Middle Triassic), eosauropterygians were already the most common fossils in the Germanic Basin. They are represented by many individuals, as well as by a number of taxa [1], [4]–[8]. Nothosaurs and pachypleurosaurs, as well as placodonts, were restricted to the Tethys, whereas pistosaurs had a global distribution [1]. The pattern of geographical distribution and the extinction of most of these early Sauropterygia (Placondontia, Pachypleurosauria, Nothosauroidea; Fig. 1A) at the end of the Middle and Late Triassic are possibly related to the disappearance of the epicontinental seas. The Plesiosauria, the sister group of Pistosauridae within Pistosauroidea, appeared during the Late Triassic. The survival and success of the Pistosauroidea is maybe related to their ability to adapt to pelagic life.

Triassic sauropterygians were part of the recovery pattern after the Permo-Triassic extinction, but after a rapid radiation and diversification most of them went extinct at the end of the Triassic. Marine reptiles can therefore greatly contribute to the understanding of major evolutionary patterns, such as recovery, extinction, and secondary aquatic adaptations.

Phylogenetic hypotheses of early Sauropterygia are the result of comprehensive studies by Rieppel and colleagues (for references see [1]; Fig. 1A). However, a study on the Chinese pachypleurosaur *Keichousaurus* [9] has recently questioned the monophyly of Pachypleurosauria (Fig. 1B). Furthermore, phylogenetic hypotheses of Triassic Sauropterygia are rather unstable, largely because most of the postcranial record of Lower Muschelkalk taxa involves isolated, unassociated material (e.g., [summarized in 1,10,11]). Additionally, postcranial morphology of early Sauropterygia is highly conservative and uniform. As a result, alpha taxonomy of Sauropterygia is solely based on skull morphology.

Early eosauropterygian humeri retain the plesiomorphic condition of their terrestrial ancestors and still exhibit a complex morphology when compared to the rest of the skeleton. Thus, humeri represent at the moment the only diagnostic postcranial element which allow, even when isolated, a taxonomic assignment at least on a group level. Eosauropterygian humeri are very common in the fossil record of the Germanic Basin and show a very diverse morphology, suggesting the existence of more taxa than those described on the basis of the cranial remains. However, little is known about ontogenetic and intraspecific variation of taxa. Sexual dimorphism, mainly expressed in humerus morphology, is documented for the pachypleurosaurs *Neusticosaurus* and *Keichousaurus* [12]–[13], but cannot be demonstrated for Lower Muschelkalk pachypleurosaurs due to the rarity of diagnostic specimens. Diversity of *Nothosaurus* humeri in the Lower Muschelkalk of the Germanic Basin was previously described [14].

Microstructure of eosauropterygian bones was described in several standard references [15]–[21]. More recently Sander [22] did a skeletochronological study of *Neusticosaurus* from the locality of Monte San Giorgio (Italy/Switzerland). Buffrénil and Mazin [23] as well as de Ricqlès and Buffrénil [24] studied bone histology of *Placodus*, and Wiffen et al. [25] described the bone histology of Cretaceous plesiosaurs. The most recent work on *Nothosaurus* and *Pistosaurus* long bone histology was done by Krahl et al. [26]. Except for the work of Sander [22] and Krahl et al. [26], most of these studies have focused on the adaptation of bone to aquatic life: pachyostosis and osteosclerosis, as well as concurrent heterochrony effects (e.g., [19], [23]–[25]). A comprehensive and comparable study on eosauropterygian bone histology has never been done. Thus, little is known about sauropterygian bone tissues and growth, and no attention has been given to the phylogenetic aspect of the observed differences.

This paper describes and compares different humeri morphotypes along with their corresponding bone histology of Lower Muschelkalk Sauropterygia, with a focus on eosauropterygians. Results uncover phylogenetically and taxonomically relevant differences in morphology and bone histology, contradicting the current phylogenetic hypothesis. Furthermore, a variety of growth patterns and bone tissues reveal unexpected insights into ontogeny, physiology, and paleobiology. Bone histology can also explain the success of the Pistosauroidea, the only eosauropterygians which survived into the Jurassic and gave rise to the plesiosaur radiation.

### Geological Background

The material studied here originates from the productive localities of Winterswijk, The Netherlands, and Freyburg, on the River Unstrut, Saxony, Germany. The limestones from the locality of Winterswijk represent the middle Lower Muschelkalk (early Anisian, Middle Triassic) and are the westernmost outcrop within the Germanic Basin. The main fossil bearing horizon is the so-called layer 9 of the quarry [27], which is part of the newly established Vossenveld Formation [28]. Layer 9 is a bonebed which mainly yields isolated bones. However, associated and at least partially articulated skeletons can be found as well, which is very atypical for the Germanic Basin. Nevertheless, sorting by water is clearly indicated by the accumulation of bones in certain places which were most likely brought in by the tide from regions farther offshore. The horizon represents a shallow marine environment consisting of massive limestones with rare traces of bioturbation and turbulences. Only *Rhizocorallum* sp., steinkerns of bivalves (cf. *Myophora*) and the shell of one brachiopod (cf. *Lingula*) are rarely preserved in layer 9 [29]. The rareness of benthic invertebrates in layer 9 points to an extreme environment nearly devoid of life. Layer 9 clearly differs from the stratigraphically somewhat older layer 4 [27], another fossil bearing horizon in the Winterswijk quarry which was interpreted as a sabkha environment [30]. Different horizons in Winterswijk clearly reflect the regression and transgression events within the Germanic Basin. Ongoing research on the marine reptile fauna in Winterswijk has so far produced three species of *Nothosaurus* [4], [6], [8], one pachypleurosaur, *Anarosaurus heterodontus* [7], [31], and placodonts [5], [32].

The rocks from Freyburg represent a continuous sequence from the uppermost Buntsandstein, through the entire Lower Muschelkalk, to the transitional level of the Middle Muschelkalk, which is characterized by the presence of evaporites. The Lower Muschelkalk profile could be described as typical Jena Formation (Wellenkalk facies) and is stratigraphically assigned to the early middle Anisian (Henniger pers. comm. 2009). The exact sequences from which the sampled bones originated is not known. They are all marked with only the locality name “Freyburg,” without any further stratigraphic comment. Although the sequence in Freyburg covers a broader stratigraphic range and represents a deeper marine facies, the sauropterygian faunal structure is similar and comparable to Winterswijk. The marine reptiles *A. heterodontus* and *N. marchicus* are described from Freyburg (summarized in [1]). *Cymatosaurus fridericianus* is known from the geographically and stratigraphically (Upper Röt Formation, early Anisian, Middle Triassic) close locality of Halle-Nietleben, Saxony, Germany [1].

### Introduction to Bone Histology

In tetrapods, primary bone tissues are formed by lamellar bone, parallel-fibered bone, or woven bone. Lamellar bone is highly organized, resulting from slow growth. Woven bone is generally deposited very quickly and accordingly is not well organized. Parallel-fibered bone displays an intermediate degree of organization and rate of deposition. In addition to its primary bone tissues, a bone type is characterized by the presence, density, and arrangement of blood vessels preserved as vascular canals in fossilized bone. During ontogeny, when growth rate decreases, bone tissue organization increases, whereas vascularization also decreases, and finally disappears when asymptotic growth is reached. The bone tissue is then avascular.

Bone tissues can be categorized into two main bone types: the lamellar-zonal bone type (LZB type) and the fibrolamellar bone type (FLB type) [33]. By definition, LZB type consists of lamellar or parallel-fibered bone [33] with only low to moderate vascularization, and bone tissue is dominated by longitudinally arranged simple vascular canals. Lamellar zonal bone deposited in the outermost cortex is called an external fundamental system (EFS) which is interpreted as the cessation of growth and thus an indication of fully grown individuals.

FLB type is the result of the sequential deposition of different bone tissues to form a complex 3D structure. In this 3D structure, woven bone is deposited very rapidly around a vascular canal and only later is the vascular canal centripetally filled in by lamellar bone, then forming a primary osteon. FLB type is generally highly vascularized and dominated by radial, laminar or irregularly arranged vascular canals.

Each of these two bone types results from a certain growth pattern. The LZB type is, because of its higher organization and lower vascularization, always produced by slow growth rates. Additionally, growth is always cyclically interrupted by growth marks. This growth pattern is generally accompanied by low metabolic rates. LZB type is always regarded as plesiomorphic, although it definitely has certain advantages such as the possibility to respond to environmental changes or instabilities within a certain developmental plasticity. This plesiomorphic growth pattern is typical for reptiles, crocodiles, and turtles (e.g., [20], [34].

The FLB type is produced by fast growth rates. Growth is often continuous and not interrupted by growth marks, such as in sauropods, large herbivorous mammals, and in most birds but can also be cyclically interrupted, as is documented for many nonavian dinosaurs. FLB type always occurs in combination with high metabolic rates. FLB type is regarded as a derived growth pattern which is typical for dinosaurs, extant birds, and large mammals [35]–[38]. However, both bone tissue types, LZB type (in form of an EFS) as well as FLB type, can occur in the same bone in certain taxa, such as in sauropodomorphs. However, as the following description will show, the current definition of bone types is not always sufficient.

The first occurrence of fibrolamellar bone dates back to the Upper Permian and was described from therapsids (e.g., [39]). Basal archosaurs such as *Erythrosuchus* already show FLB type [17], [40] as do the first dinosaurs (e.g., [41], [42]). In Sauropterygia fibrolamellar bone was formerly known only from Cretaceous plesiosaurs [25]. However, the current work documents the presence of fibrolamellar bone in the most basal sauropterygians, as old as the early Anisian (Middle Triassic), and at the origin of the group. Thus, FLB type or, respectively, fibrolamellar bone is known in three major vertebrate clades: in archosaurs (including birds, dinosaurs, pterosaurs, and possibly crocodiles), in the therapsid clade (including mammals), and in sauropterygians. It is assumed that fibrolamellar bone developed convergent in each group.

## Results

### General Humerus Morphology of Eosauropterygia

Early sauropterygian humeri can be classified into three main groups: the “placodont” type, the “nothosaur” type and the “pachypleurosaur” type. The nearly half-rounded placodont type exhibits a rather simple morphology and is easy to distinguish from the “nothosaur” type and the “pachypleurosaur” type. The latter two both have in common a smooth and oblate ventral side. A prominent deltopectoral crest is not developed, but in older individuals it is indicated by a rough and rugose area. The latissimus dorsi insertion is indicated by a ridge on the dorsal side of the proximal end. The proximal head can have an oblong articulation surface.

**Figure 2:**
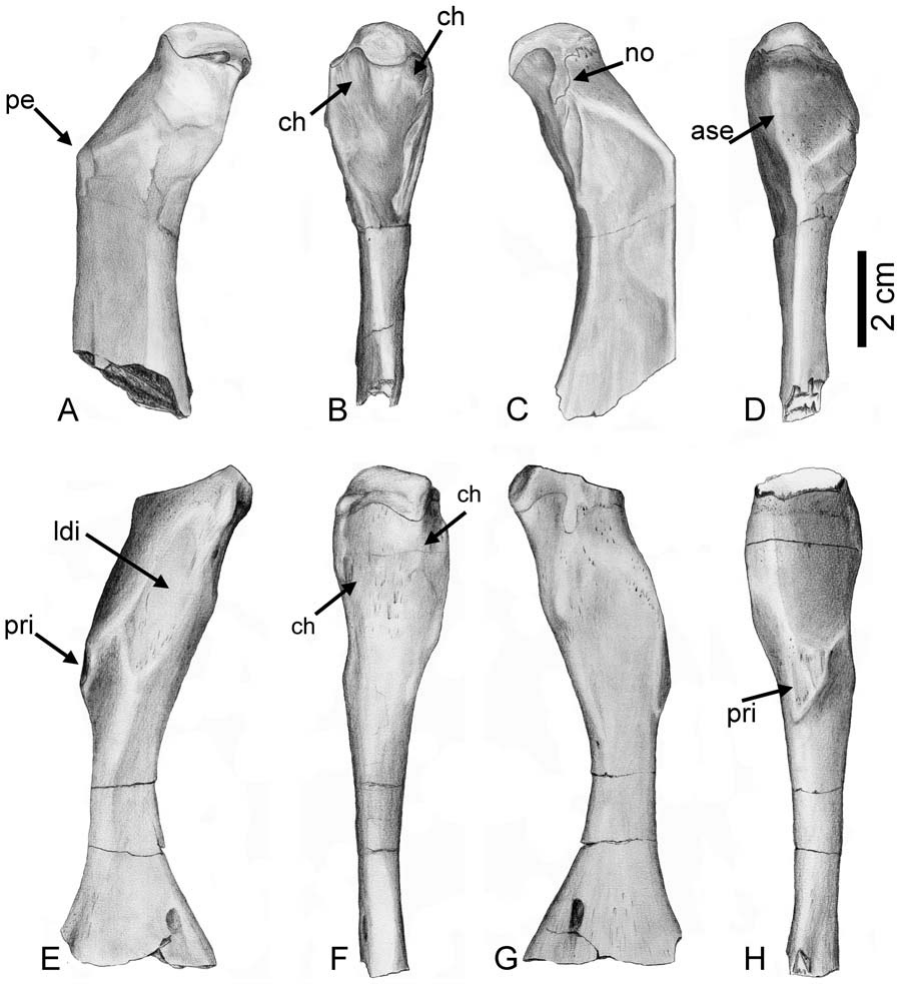
Drawing of *Nothosaurus* humerus morphotype IV and *Cymatosaurus* morphotype I. A–D) Horizontally mirrored drawing of *Nothosaurus* humerus morphotype IV (right humerus IGWH-8). A) Dorsal view, B) postaxial view, C) ventral view, and D) preaxial view. E–H) Drawing of *Cymatosaurus* humerus morphotype I (left humerus IGWH-19). E) Dorsal view, F) postaxial view, G) ventral view, and H) preaxial view. Abbreviations in alphabetic order: ase  =  asymmetrically sharp edge which divides the preaxial proximal surface into two postaxially descending surfaces; ch  =  deep channel; ldi  =  latissimus dorsi insertion, no  =  distinct strangulated notch; pe  =  protruding edge; pri  =  prominent rhombic indentation. The deep channels are not easily visible in 2F but the arrows mark their position.

**Figure 3:**
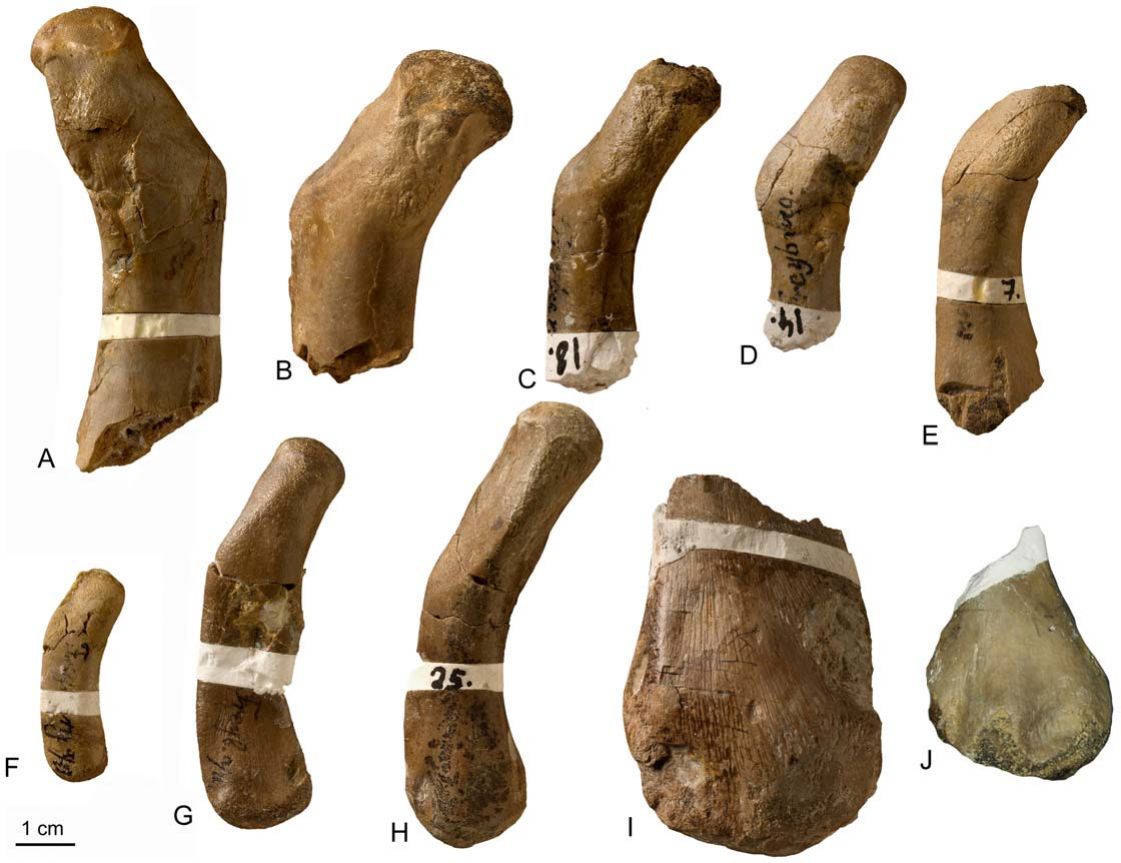
*Nothosaurus* humeri from Freyburg locality in dorsal view. A–E) *Nothosaurus* humerus morphotype IV. A) Right humerus IGWH-8, B) left humerus IGWH-30, C) left humerus IGWH-18, D) left humerus IGWH-14, and E) left humerus IGWH-7, F) Left humerus IGWH-3. G–H) *Nothosaurus* humerus morphotype II. G) Right humerus IGWH-28, and H) left humerus IGWH-25. I) Right humerus IGWH-4. J) Left humerus IGWH-17. IGWH-3, -4, and -17 are not assigned to a *Nothosaurus* humerus morphotype. White plaster marks sampling locations.

**Figure 4:**
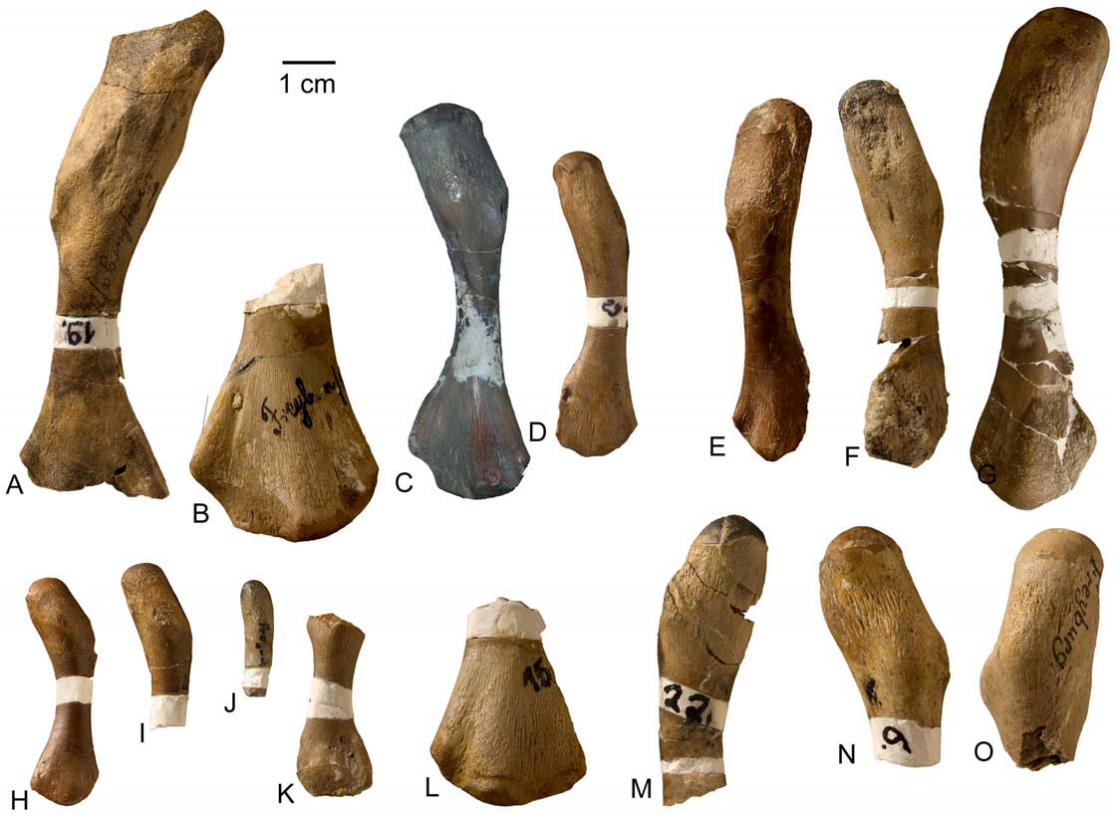
*Cymatosaurus* humeri morphotype I and II, and unassigned “pachypleurosaur” humeri showing histotype A bone tissue from Freyburg and Winterswijk localities in dorsal view. A–D) “Pachypleurosaur” humerus morphotype I. A) Left humerus IGWH-19, B) right humerus IGWH-20, C) right humerus NMNHL RGM 449487, which is the counterpart of the sampled left humerus (IGWH-19), and D) right humerus IGWH-29. The arrow in B and C marks the proximally shifted entepicondyle characteristic for this morphotype. E–G) “Pachypleurosaur” humerus morphotype II. E) Left humerus NMNHL ST 445912, F) right humerus IGWH-1, and G) left humerus IGWH-26/27. H) Right humerus IGWH-11. I) Right humerus IGWH-12. J) Right humerus IGWH-16. K) Left humerus IGWH-10. L) Left humerus IGWH-15. M) Left humerus IGWH-22. N) Right humerus IGWH-6. O) Left humerus IGWH-31. The humeri illustrated in samples H–O are not assigned to a “pachypleurosaur” humerus morphotype. White plaster marks sampling locations.

**Figure 5:**
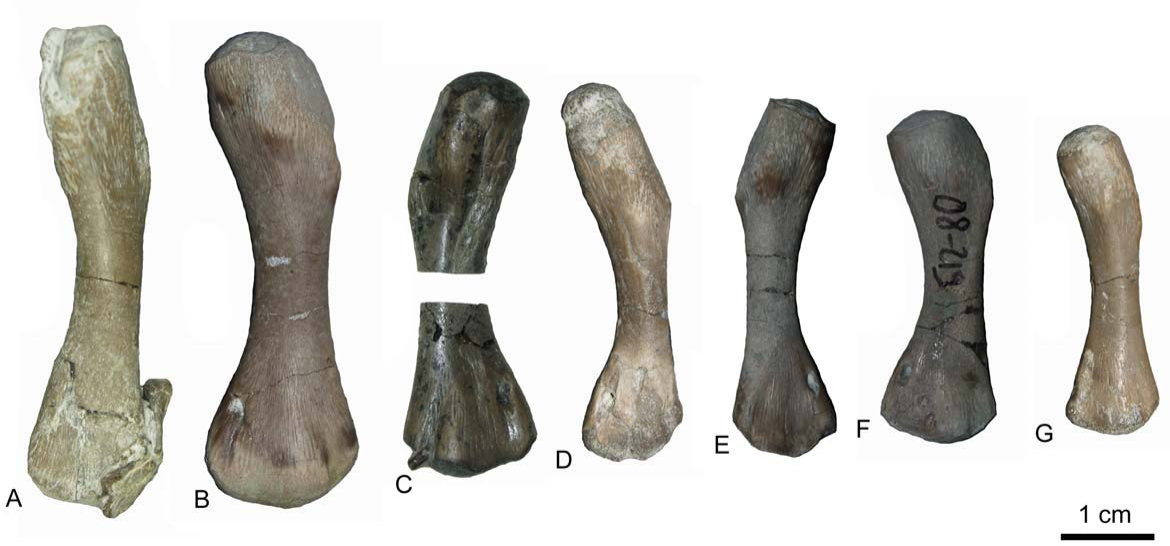
*Anarosaurus heterodontus* humeri from Winterswijk in dorsal view. A) Left humerus Wijk08-183. B) Right humerus Wijk09-58. C) Left humerus Wijk09-472. D) Right humerus Wijk07-50. E) Left humerus Wijk09-543. F) Right humerus Wijk08-219. G) Right humerus Wijk07-137. Except for Wijk09-472, specimens were figured before sampling.

In the “nothosaur” type, the preaxial proximal side is asymmetrically divided into two postaxially descending surfaces (Fig. 2D). The dorsal surface is always larger than, but not as steep as, the ventral one. In the “pachypleurosaur” type, the preaxial proximal side forms a planar, nearly rectangular surface (Fig. 2H). In some specimens, this surface slightly descends towards the dorsal side, but is not as asymmetrical as in the “nothosaur” type. In older individuals the postaxial proximal side is marked by two deep channels running dorsally and ventrally down from the proximal head (Fig. 2B, F). The length of the proximal end, in relation to the length of the shaft, can vary in both types (Figs. 3, 4 and 5). The bone surface of the “pachypleurosaur” type is generally more textured than that of the “nothosaur” type. Additionally, the “pachypleurosaur” type carries distinct striations on its preaxial proximal end (Figs. 4 and 5).

Both humeri types are easy to distinguish on the basis of their shaft region: in the “pachypleurosaur” type the shaft region is always constricted and clearly set off from the proximal and distal end. In particular, the distal end broadens distinctly and sometimes is even fan-shaped. The cross section of the midshaft is round to oval in the “pachypleurosaur” type. In the “nothosaur” type, the shaft is not constricted and is triangular in cross section, as a result of their distinctly thin preaxial shaft margin. The preaxial shaft margin in the “nothosaur” type is straight, contrasting with their slightly concave postaxial shaft margin. The “pachypleurosaur” type carry a rhombic indentation ventrally on the preaxial proximal shaft, which is most likely the attachment site of the pectoralis muscle (Fig. 2H). This indentation is not observed in the “nothosaur” type. The “nothosaur” type carries postaxially a foramen in the middle of the proximal shaft. This foramen is also visible in some specimens of the “pachypleurosaur” type (IGWH-1, IGWH-10, IGWH-19, IGWH-29, Wijk07-137, Wijk08-183, Wijk09-58).

In addition to its fan-shape, the overall morphology of the distal end of the “pachypleurosaur” type is more complex when compared to the “nothosaur” type (Figs. 2, 3, 4 and 5) [43]. The “pachypleurosaur” humerus is generally more complex and more closely resembles the plesiomorphic (terrestrial) condition. The only solid indication of a mature ontogenetic stage in both groups is the shaping of the morphological characteristics. The “nothosaur” type is assigned to *Nothosaurus* whereas the “pachypleurosaur” type comprises at least two eosauropterygian groups, as histology will show.

### General Comments on Eosauropterygian Long Bone Histology

#### Primary Cortex

**Figure 6:**
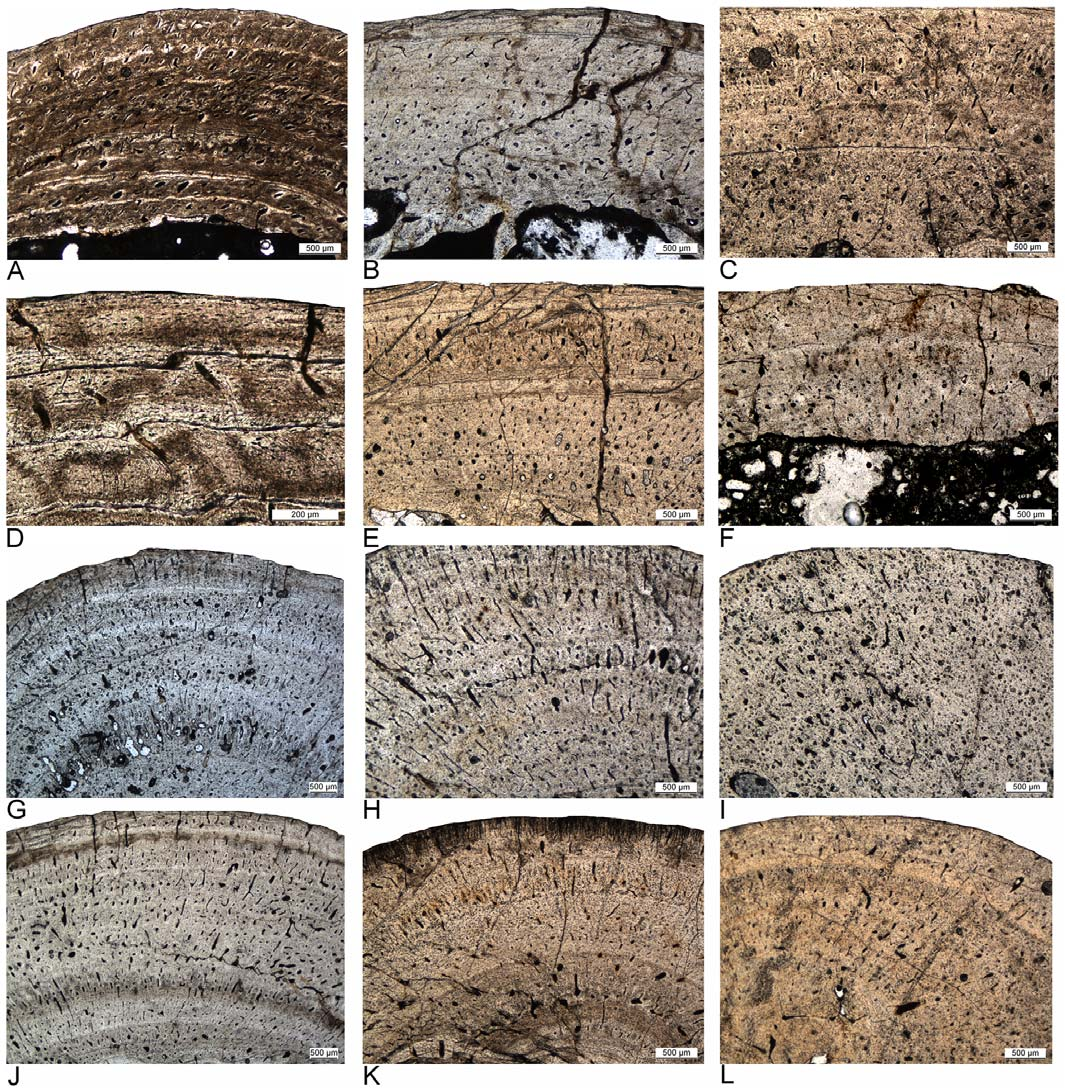
Different vascular density in humeri of adult *Nothosaurus* and *Cymatosaurus* (histotype A). A–D) Samples of *Nothosaurus* morphotype IV humeri in normal light. Vascular density decreases from A) to C). A) Relatively high vascular density in IGWH-7, preaxial bone side. B) Moderate vascular density in IGWH-18, postaxial bone side. C) Low vascular density in IGWH-8, postaxial bone side, and D) enlargement of avascular lamellar zonal bone in IGWH-8, ventral bone side. Note in D) also the high number of flat osteocytes in the nearly avascular tissue. E–F) Samples of *Nothosaurus* morphotype II humeri in normal light. Vascular density decreases from E) to F). E) Relatively high vascular density in IGWH-25, preaxial bone side. F) Only moderate vascular density in IGWH-28, postaxial bone side. G–L) Samples of *Cymatosaurus* (histotype A) in normal light. Vascular density decreases from G) to L). G) High vascular density in IGWH-6, preaxial bone side. H) High vascular density in IGWH-1, ventral bone side. I) High vascular density in NMNHL RGM 449487, postaxial bone side. J) Moderate vascular density in IGWH-20, ventral bone side. K) Moderate to low vascular density in IGWH-19, preaxial bone side. L) Low vascular density in IGWH-29, preaxial bone side. Please note also the differences in growth marks. Whereas nothosaur humeri (A–F) have lines of arrested growth (LAGs) developed, histotype A samples (G–L) show mainly annuli.

**Figure 7:**
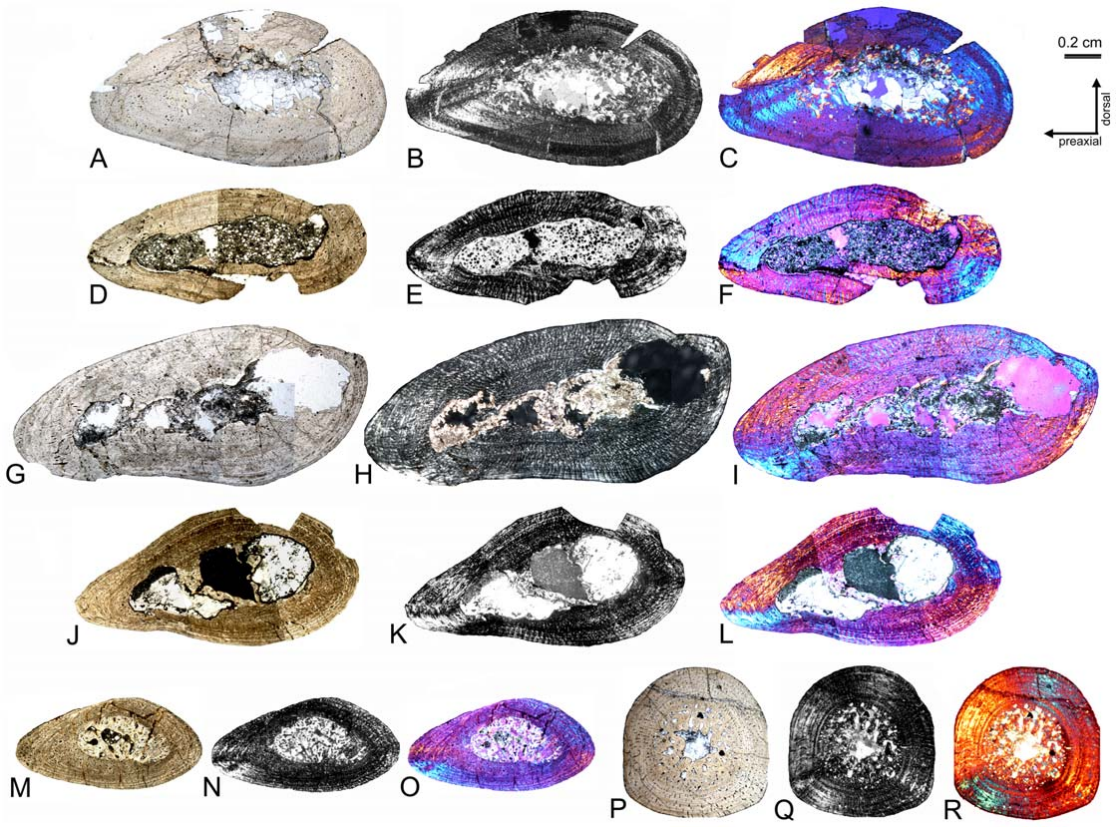
Cross sections of *Nothosaurus* humeri. A–F) Cross sections of *Nothosaurus* humerus morphotype II. A) Composite of *Nothosaurus* humerus IGWH-25 in normal light, B) polarized light, and C) polarized light combined with gypsum filter. D–E) Composite and mirrored cross section of *Nothosaurus* humerus IGWH-28 in D) normal light, E) polarized light, and F) polarized light combined with gypsum filter. Please note the vesicles throughout the entire medullary cavity. G–L) Cross sections of *Nothosaurus* humerus morphotype IV. G–I) Composite and mirrored cross section of *Nothosaurus* humerus IGWH-8 in G) normal light, H) polarized light, and I) polarized light combined with gypsum filter. J–L) Composite of *Nothosaurus* humerus IGWH-18 in J) normal light, K) polarized light, and L) polarized light combined with gypsum filter. M–N) Cross section of nothosaur humerus IGWH-3 in M) normal light, N) polarized light, and O) polarized light combined with gypsum filter. P–Q) Cross section of nothosaur femur Wijk05-10 in P) normal light, Q) polarized light, and R) polarized light combined with gypsum filter.

**Figure 8:**
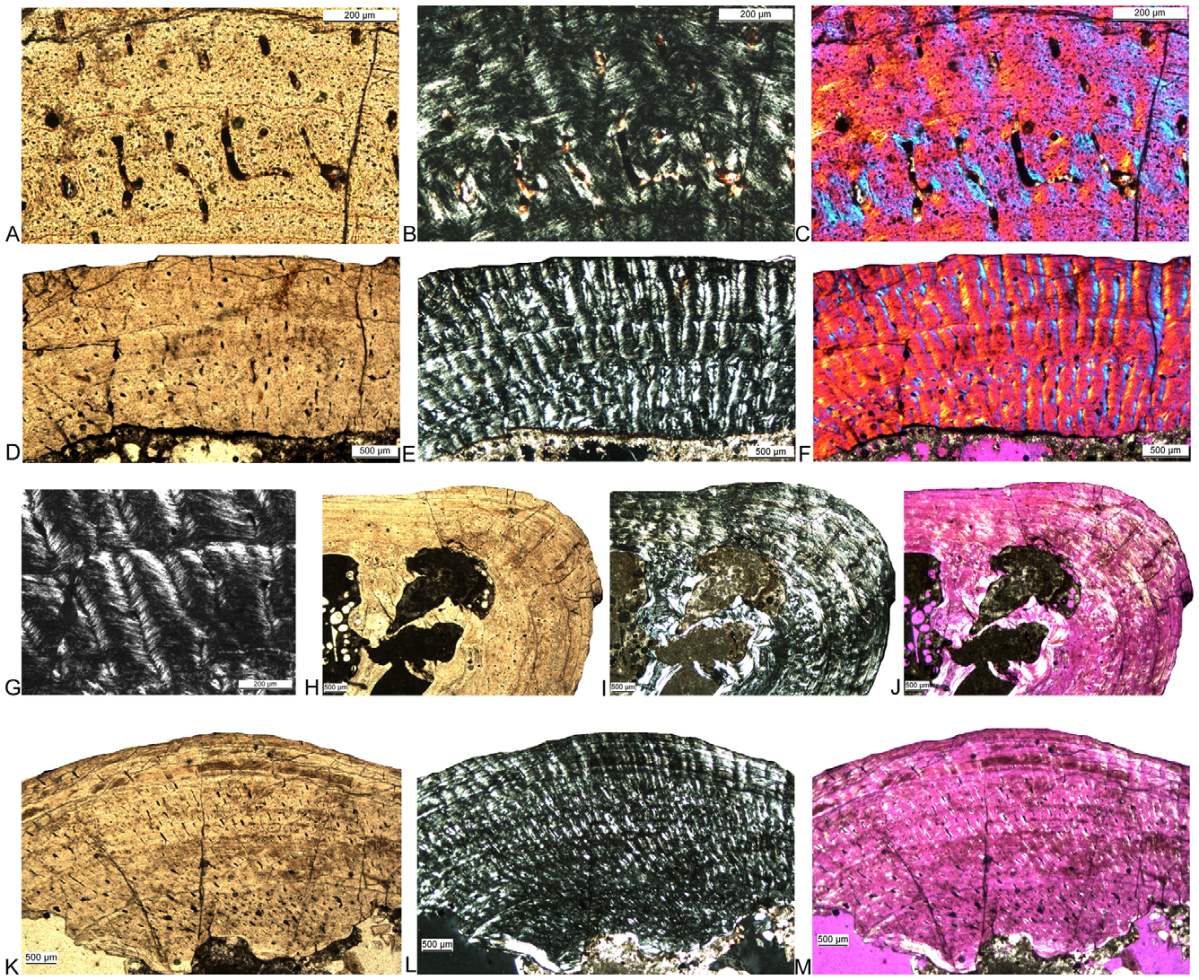
Histological details of *Nothosaurus* long bones. A–C) Fibrolamellar bone in *Nothosaurus* femur Wijk05-10, ventral bone side. A) Normal light, B) polarized light, and C) polarized light combined with gypsum filter. D–F) Typical *Nothosaurus* humerus morphotype II bone tissue and growth pattern in IGWH-28, postaxial bone side. D) Normal light, E) polarized light, and F) polarized light combined with gypsum filter. G) Funnel-shaped vascular canals in polarized light, detail of IGWH-28. H–J) Lamellar zonal bone in the outer cortex of IGWH-17, preaxial bone side. H) Normal light, I) polarized light, and J) polarized light combined with gypsum filter. K–M) Typical *Nothosaurus* morphotype IV bone tissue and growth pattern in IGWH-8, ventral bone side. K) Normal light, L) polarized light, and M) polarized light combined with gypsum filter.

Parallel-fibered bone is the dominant bone tissue in the “nothosaur” humerus type, with a greater amount of woven bone in younger individuals and an increasing amount of lamellar bone in older individuals. In *Nothosaurus*, the vascular density is low, and even in younger individuals it is only moderate when compared to the pachypleurosaur histotypes (Figs. 6, 7 and 8). The inner cortex, as well as the thin cortex of the ventral and dorsal side, is dominated by longitudinal canals. If present, radial canals are concentrated in the preaxial and postaxial cortex. In older individuals, some primary osteons can develop. *Nothosaurus* humeri often have a funnel-shaped arrangement of the crystallites around simple vascular canals (Fig. 8E–G) which can also occur in some humeri of the “pachypleurosaur” type, but they are less common and less distinct here. The preaxial and postaxial cortex generally contains a greater amount of lamellar bone than the ventral and dorsal cortex. Small layers of fibrolamellar bone can occur locally in older individuals (Fig. 8A–C) but remain rare and are not seen in every specimen. Development of an EFS has only been observed in one *Nothosaurus* specimen (IGWH-17; Fig. 8H–J). The cortex is generally thicker dorsopreaxially than ventropostaxially. Growth in *Nothosaurus* shows an increasing organization of bone tissue and simultaneously a decreasing vascular density from the inner to the outer cortex. The cortex is regularly interrupted by growth marks, mainly in form of LAGs (lines of arrested growth) (Figs. 6, 7 and 8). The distance between these growth marks is irregular, although it becomes gradually smaller towards the outer cortex.

**Figure 9:**
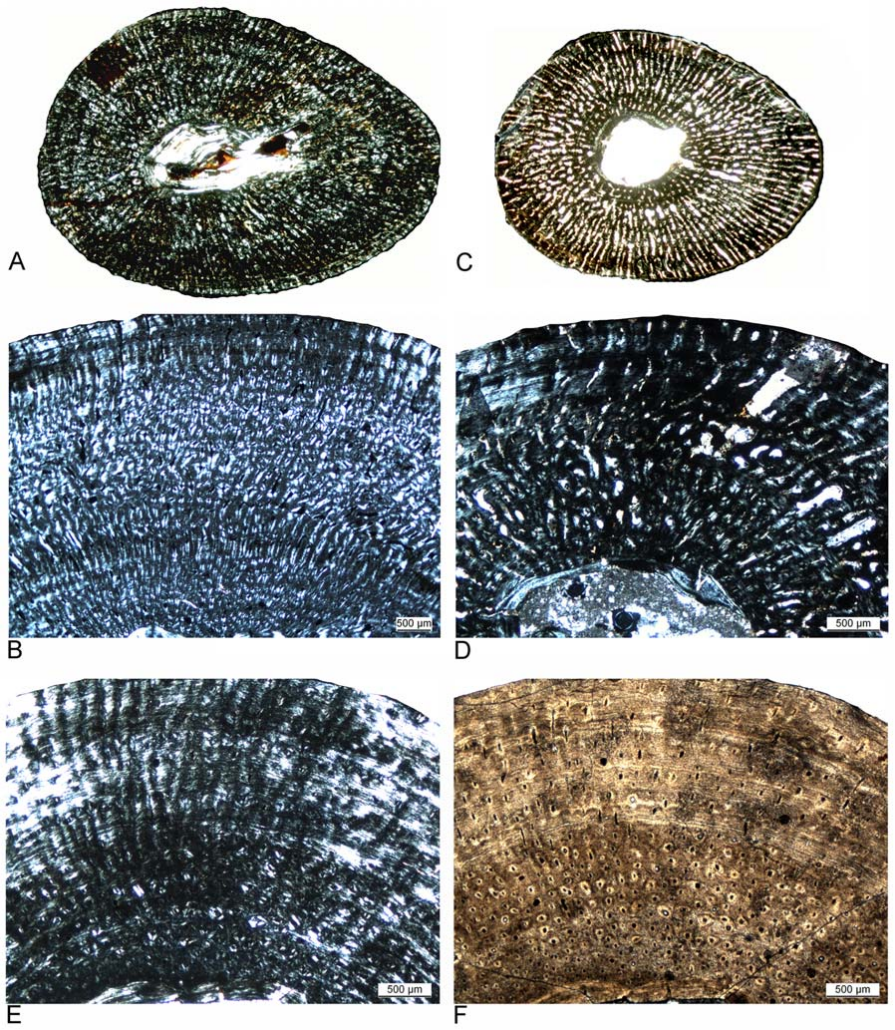
Differences in bone tissue between *Cymatosaurus* (histotypes A) and *Anarosaurus heterodontus* (histotype B). A–B) Humerus showing histotype A bone tissue in A) a juvenile (IGWH-16) and B) an adult (IGWH-20, ventral bone side) individual, both in polarized light. C–D) Humeri showing histotype B bone tissue in C) a juvenile (Wijk08-543) and D) an adult (Wijk08-58, preaxial bone side) individual, both in polarized light. Note the difference in vascular density and tissue organization between histotypes A and B. E–F) Femur showing histotype A bone tissue in an adult individual (IGWH-24, dorsal bone side) in E) polarized and F) normal light. Contrary to histotype A humeri, the inner cortex of femora showing histotype A bone tissue is dominated by large longitudinal vascular canals.

**Figure 10:**
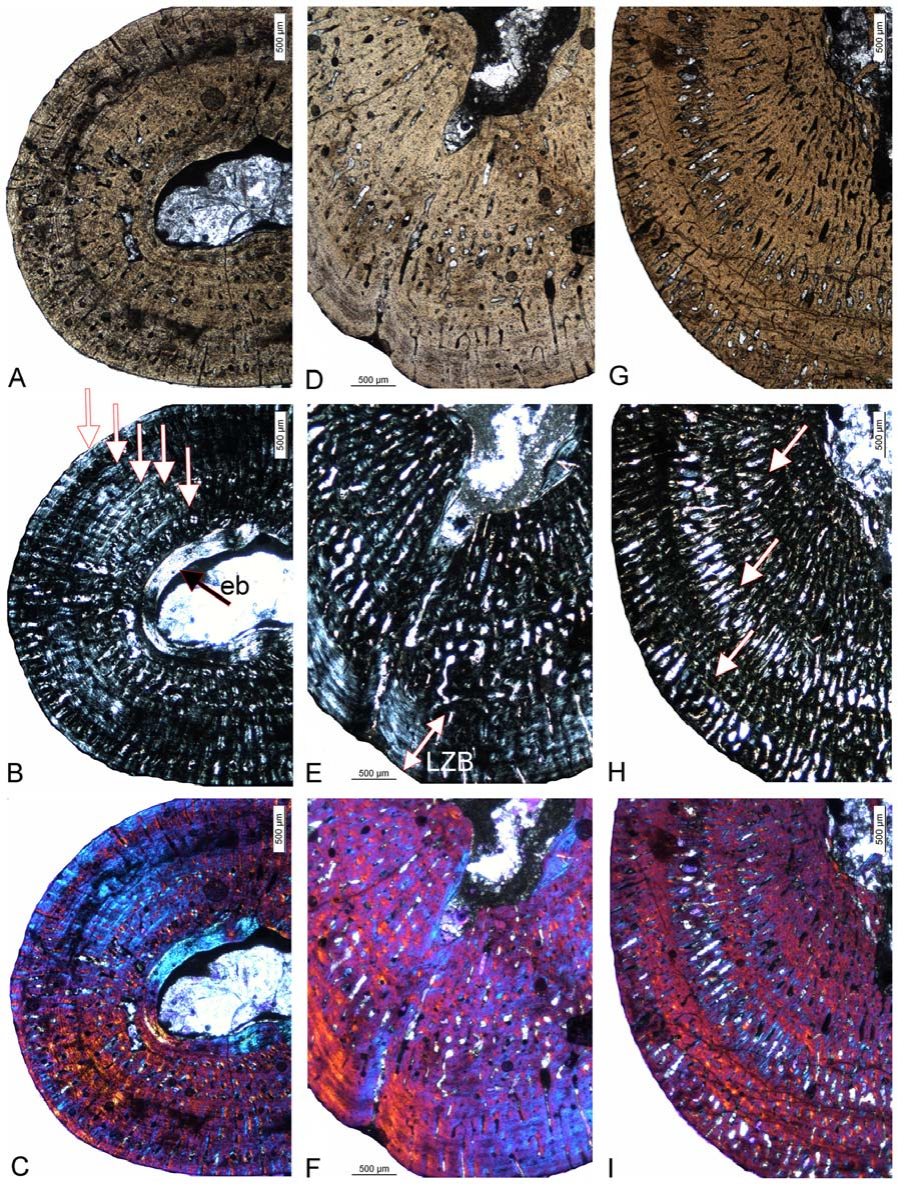
Histological subtypes of *Anarosaurus heterodontus* humeri (histotype B). A–C) Histological subtype of histotype B with the cortex regularly stratified by annuli. Enlargement of the preaxial half of humerus Wijk07-50 in A) normal, B) polarized light, and C) polarized light combined with gypsum filter. The black arrow in B) marks the well developed layer of endosteal bone (eb) and the white ones mark growth marks. D–F) Histological subtype of histotype B with the internal cortex continuously vascularized until bone tissue changes to lamellar zonal bone in the outer cortex. Enlargement of the ventropostaxial half of humerus Wijk09-58 in D) normal, E) polarized light, and F) polarized light combined with gypsum filter. The arrow in E) marks the outer layer of lamellar zonal bone (LZB). G–I) Histological subtype of histotype B with the vascular canals arranged in rows. Enlargement of the ventropostaxial half of humerus Wijk08-183 in G) normal light, H) polarized light, and I) polarized light combined with gypsum filter. The arrows in H) mark the changes in bone tissue due to the arrangement of vascular canals in rows. These changes are not accompanied by distinct growth marks.

**Figure 11:**
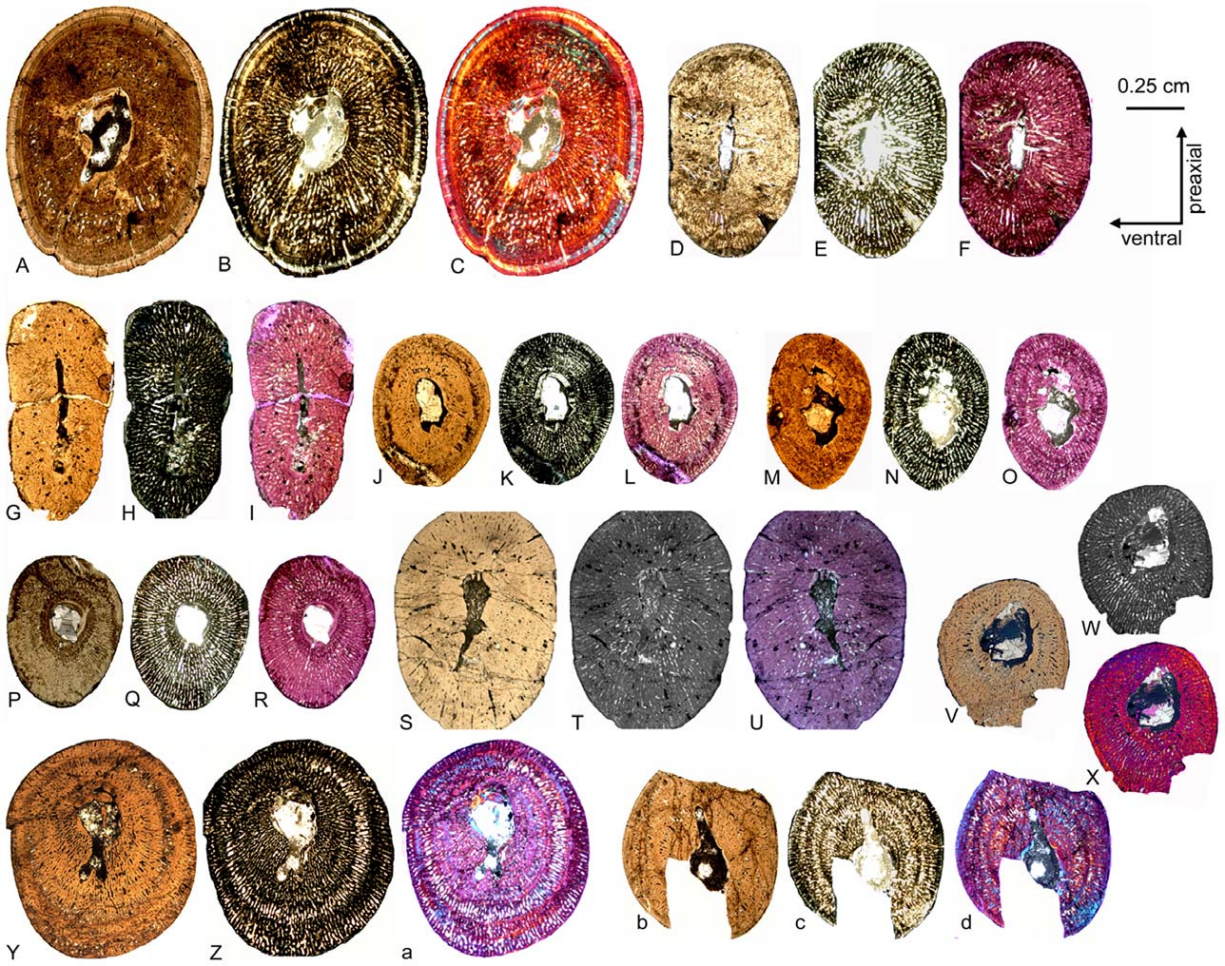
Cross sections of *Anarosaurus heterodontus* humeri (histotype B). A–I) Subtype of histotype B bone tissue with the internal cortex showing continuously vascularized until a sudden change to lamellar zonal bone in the outer cortex. A–C) Cross section of humerus Wijk09-58 in A) normal light, B) polarized light, and C) polarized light combined with gypsum filter. D–F) Cross section of humerus Wijk08-219 in D) normal light, E) polarized light, and F) polarized light combined with gypsum filter. G–I) Horizontally mirrored cross section of humerus Wijk06-238 in G) normal light, H) polarized light, and I) polarized light combined with gypsum filter. J–U) Subtype of histotype B bone tissue with the inner cortex regularly stratified by annuli. J–L) Cross section of humerus Wijk07-50 in J) normal light, K) polarized light, and L) polarized light combined with gypsum filter. M–O) Horizontally mirrored cross section of humerus Wijk07-137 in M) normal light, N) polarized light, and O) polarized light combined with gypsum filter. P–R) Cross section of humerus Wijk09-543 in P) normal light, Q) polarized light, and R) polarized light combined with gypsum filter. S–U) Cross section of humerus Wijk09-472 in S) normal light, T) polarized light, and U) polarized light combined with gypsum filter. V-d) Subtype of histotype B bone tissue with vascular canals arranged in rows. V–X) Horizontally mirrored cross section of humerus NME48000085c in V) normal light, W) polarized light, and X) polarized light combined with gypsum filter. Y-a) Vertically mirrored cross section of humerus Wijk08-183 in Y) normal light, Z) polarized light, and a) polarized light combined with gypsum filter. b-d) Cross section of humerus Wijk07-70 in b) normal light, c) polarized light, and d) polarized light combined with gypsum filter.

**Figure 12:**
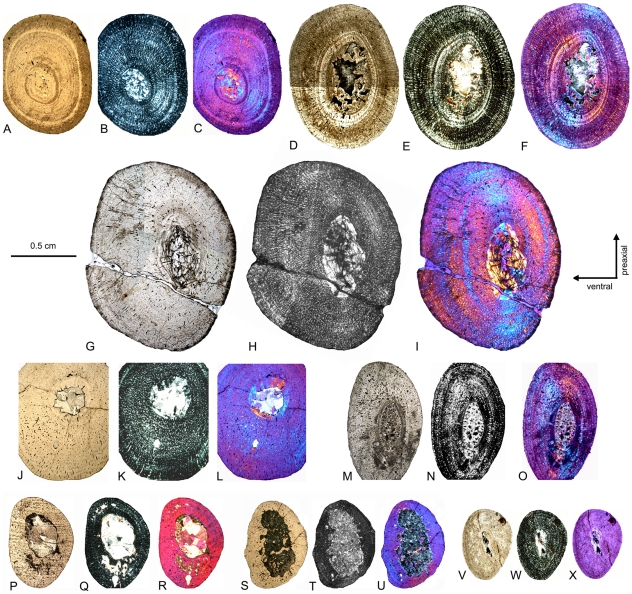
Cross sections of *Cymatosaurus* humeri (histotype A). A–C) Cross section of humerus IGWH-29 in A) normal light, B) polarized light, and C) polarized light combined with gypsum filter. D–E) Cross section of humerus IGWH-1 in D) normal light, E) polarized light, and F) polarized light combined with gypsum filter. G–I) Composite and horizontally mirrored cross section of humerus IGWH-19 in G) normal light, H) polarized light, and I) polarized light combined with gypsum filter. J–L) Cross section of humerus NMNHL RGM 449487 in J) normal light, K) polarized light, and L) polarized light combined with gypsum filter. M–O) Cross section of humerus IGWH-10 in M) normal light, N) polarized light, and O) polarized light combined with gypsum filter. P–R) Horizontally mirrored cross section of humerus IGWH-11 in P) normal light, Q) polarized light, and R) polarized light combined with gypsum filter. S–U) Horizontally mirrored cross section of humerus IGWH-12 in S) normal light, T) polarized light, and U) polarized light combined with gypsum filter. V–X) Horizontally mirrored cross section of humerus IGWH-16 in V) normal light, W) polarized light, and X) polarized light combined with gypsum filter.

**Figure 13:**
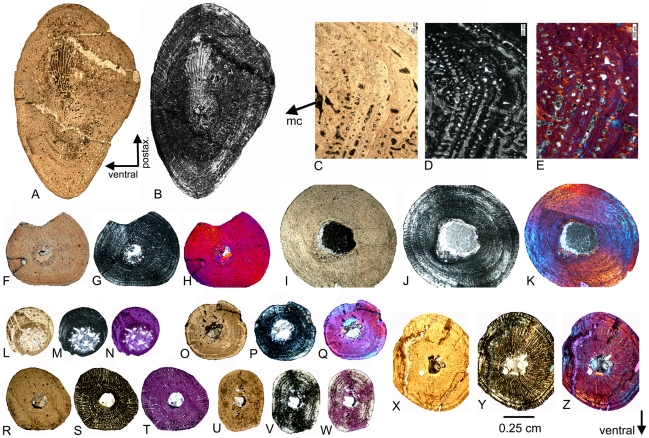
Bone tissue of placodonts, and femur cross sections showing histotype A and B bone tissue. A–B) Composite of cross section of placodont humerus IGWH-9 in A) normal and B) polarized light. C–E) Enlargement of the dorsal side of the cross section of placodont femur IGWH-23 in C) normal, D) polarized light, and E) polarized light combined with gypsum filter. The arrow in C) marks the beginning and direction of the diffuse medullary cavity. F–Q) Femur cross sections showing histotype A bone tissue. F–H) Cross section of femur NME48000074 in F) normal light, G) polarized light, and H) polarized light combined with gypsum filter. I–K) Composite of femur IGWH-24 in I) normal light, J) polarized light, and K) polarized light combined with gypsum filter. L–N) Cross section of femur IGWH-2 in L) normal light, M) polarized light, and N) polarized light combined with gypsum filter. O–Q) Cross section of femur IGWH-5 in O) normal light, P) polarized light, and Q) polarized light combined with gypsum filter. R–Z) Femur cross sections showing histotype B bone tissue. R–T) Cross section of femur Wijk-A568 in R) normal light, S) polarized light, and T) polarized light combined with gypsum filter. U–W) Cross section of femur Wijk-06-102 in U) normal light, V) polarized light, and W) polarized light combined with gypsum filter. X–Z) Cross section of femur Wijk-08-150 in X) normal light, Y) polarized light, and Z) polarized light combined with gypsum filter. Orientation in IGWH-2 and Wijk-A568 is uncertain. Wijk08-150 and Wijk06-102 are laterally compressed.

**Figure 14:**
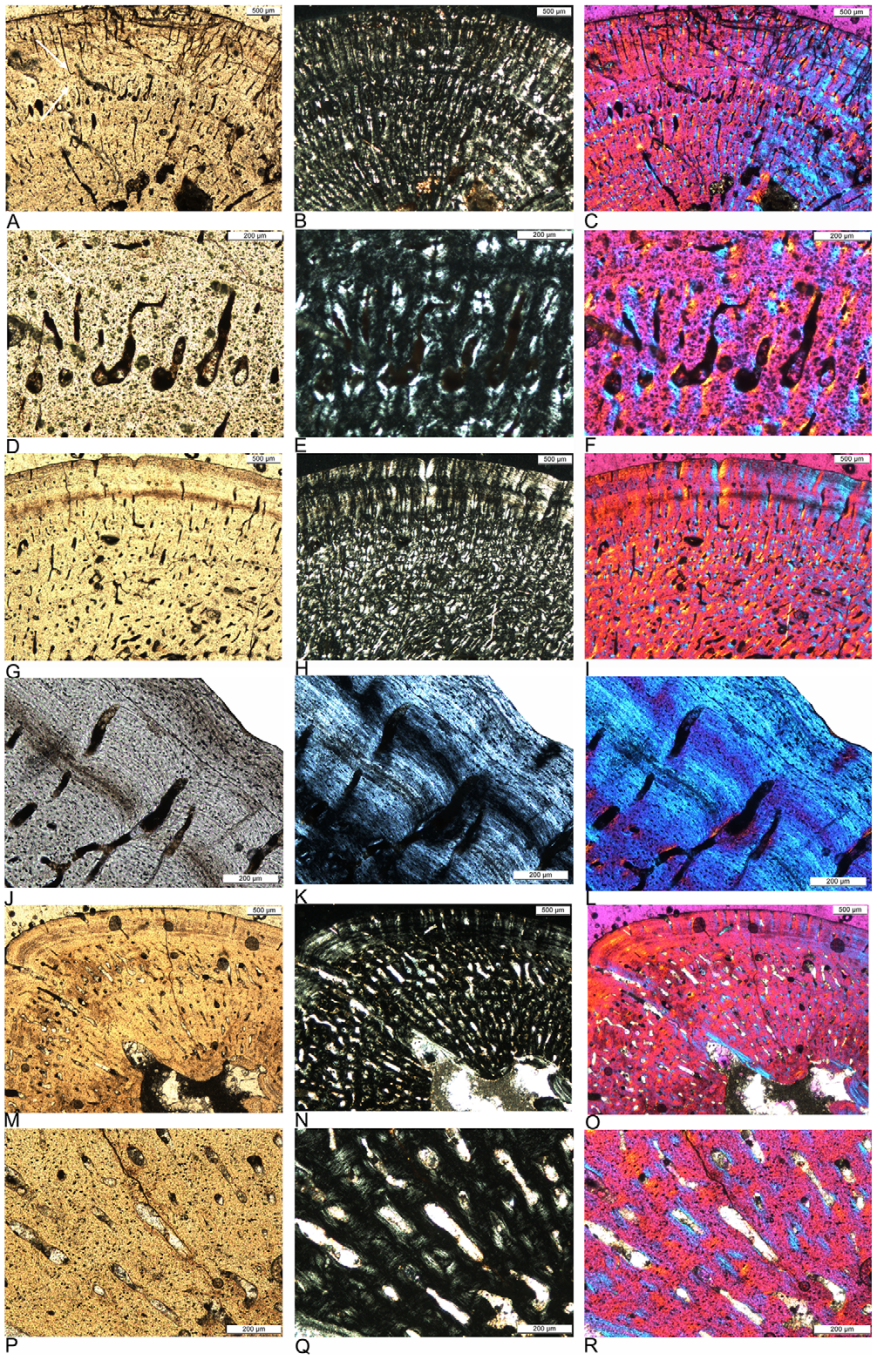
Histological details of *Cymatosaurus* (histotype A) and *Anarosaurus heterodontus* (histotype B) humeri. A–C) Typical histotype A bone tissue and growth pattern in IGWH-1, postaxial bone side, in A) normal light, B) polarized light, and C) polarized light combined with gypsum filter Arrows mark the fibrolamellar bone layer. D–F) Enlarged detail of fibrolamellar bone in the same sample, in D) normal light, E) polarized light, and F) polarized light combined with gypsum filter. G–I) Typical histotype A bone tissue and growth pattern in an adult individual, IGWH-20, ventral bone side, in G) normal light, H) polarized light, and I) polarized light combined with gypsum filter J–L) Enlarged detail of lamellar zonal bone in the same sample, in J) normal light, K) polarized light, and L) polarized light combined with gypsum filter. M–O) Typical histotype B bone tissue and growth pattern in Wijk09-58, postaxial bone side = , in M) normal light, N) polarized light, and O) polarized light combined with gypsum filter. P–R) Enlarged detail of fibrolamellar bone in the same sample, in P) normal light, Q) polarized light, and R) polarized light combined with gypsum filter.

The histology of “pachypleurosaur” humeri and femora from the Lower Muschelkalk differs from that of *Nothosaurus* in three main features. First, “pachypleurosaur” long bones have a very high vascular density which is dominated by a radial arrangement of vascular canals (Figs. 6, 9, 10, 11, 12, 13 and 14). Second, layers of fibrolamellar bone intercalated in the parallel-fibered bone tissue regularly occur, or most of the cortex consists of incipient fibrolamellar bone, which means that parallel-fibered bone tissue contains a high amount of woven bone, but not all vascular canals have been transformed into primary osteons (Figs. 6, 9, 10, 14). Both occurrences of fibrolamellar bone are independent of ontogenetic stage. Third, numerous large and thick osteocytes characterize the entire cortex of “pachypleurosaurs,” even in layers containing high amounts of lamellar bone (Fig. 14J–L). Development of lamellar zonal bone tissue, presumably representing an EFS, has only been observed in a few samples (e.g., IGWH-20; Wijk09-58; Figs. 10D–F, 14G–L).

**Figure 15:**
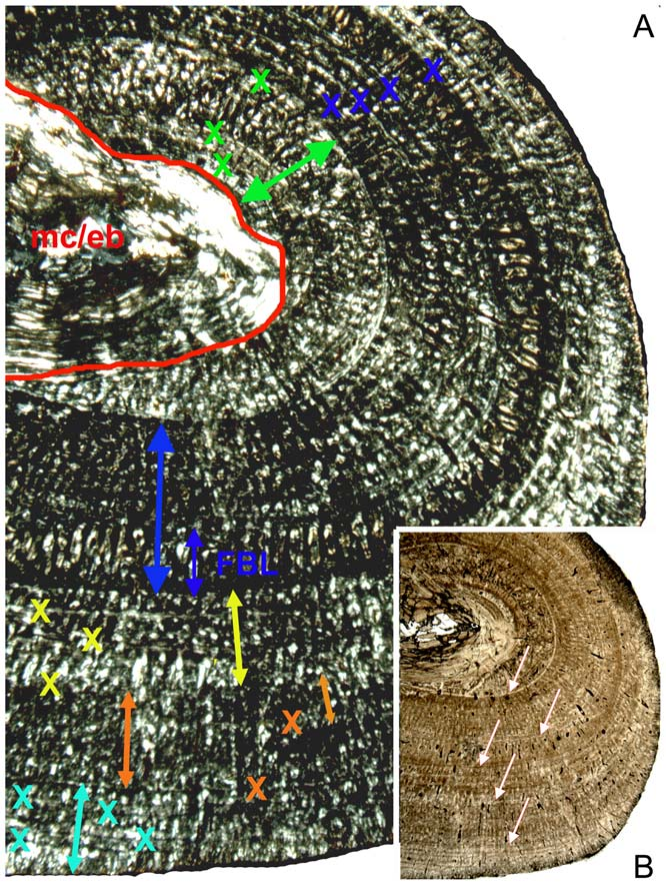
Growth pattern of *Cymatosaurus* (histotype A). A) Postaxial bone side of humerus IGWH-19 in polarized light. The red line marks the sharp border from the by endosteally (eb) filled medullary cavity (mc) to the primary cortex. The following arrows mark the 5 main growth phases. The green arrow marks the inner phase of moderate growth. Two annuli are already developed within this inner phase of moderate growth. The large blue arrow marks the following phase of fast growth containing a thick layer of fibrolamellar bone (small blue arrow). The yellow arrow marks a phase of slow growth, consisting of well organized parallel-fibered bone tissue. The orange arrow marks a second phase of fast growth, again containing a layer of fibrolamellar bone (small orange arrow). The turquoise arrow marks another phase of slow growth. Each X marks an annulus. B) Same sample in normal light. Arrows mark the main LAGs, traceable in normal light throughout the entire cross section. Under this magnification and at this bone side are only 5 LAGs visible, whereas the same clipping under polarized light reveals altogether 16 growth marks (annuli and LAGs).

The “pachypleurosaur” long bone sample can be divided into two histological groups, referred to in the following as histotype A and histotype B (Fig. 9). In both histotypes, the bone tissue is dominated by a radial pattern of vascularization. However, vascular density is distinctly lower in histotype A than in histotype B, although still much higher than in *Nothosaurus* (Figs. 6, 9, 10). Histotype A bone tissue is dominated by a radial vascular pattern but also high amounts of longitudinal vascular canals occur (Figs. 6, 9). On average, vascular canals take up between 5% to 10% in histotype A bone tissue. Some primary osteons formed already in young individuals and their number increased during ontogeny. Parallel-fibered bone tissue dominates, but distinct layers of fibrolamellar bone are regularly intercalated in the middle and outer cortex. Samples of histotype A show an iterating change in bone tissue and vascular organization which stratifies the cortex into alternating growth phases (e.g., Figs. 9, 12, 14A–C, 14G–I, 15; Table S1). The growth record always starts with an initial phase of only moderate growth, indicated by parallel-fibered bone tissue dominated by longitudinal canals (green arrow in Fig. 15). The thickness of this initial layer, as well as of all the following growth phases, can vary due to individual growth history and ontogenetic stage. In many samples, the dorsal cortex shows a high amount of lamellar bone in this initial moderate growth phase or already contains here several annuli (Fig. 15). In samples proximal and distal to the midshaft the initial phase is not preserved, leaving an incomplete growth record. This initial growth phase is well separated from the younger bone tissue by changes in vascular canal and bone tissue organization, as well as by one or two closely spaced LAGs (Figs. 6J, 15). The following growth phase is dominated by radial canals and contains layers of fibrolamellar bone. This phase of faster growth is also regularly stratified by diffuse annuli as well as by changes in vascular canal and bone tissue organization (blue arrow in Fig. 15). It usually terminates in another growth mark, often a LAG. In many histotype A samples, the outer cortex shows an increase in organization, produced by a high amount of lamellar bone and an increasing dominance of longitudinal canals (yellow arrow in Fig. 15). However, a second phase of faster growth follows again in some specimens (e.g., IGWH-19, IGWH-20; orange arrow in Fig. 15). It is indicated again by poorly organized parallel-fibered bone tissue with intercalated layers of fibrolamellar bone and a high radial vascular density. This second phase of fast growth is again followed by a phase of slow growth (turquoise arrow in Fig. 15) which finally ends in an EFS. Within the histotype A sample, only IGWH-20 shows lamellar zonal bone tissue in the outer cortex, presumably representing an EFS. However, this tissue also is repeatedly interrupted by phases of faster growth (Fig. 14G–L). Aside from the few distinct LAGs separating the main growth phases, annuli are present in histotype A throughout the entire cortex, but they remain very diffuse and are not traceable through the entire cross section.

Histotype B is also characterized by a cortex dominated by radial canals (Figs. 9, 10, 11 and 13). Individual vascular canals are very large, but their total amount is also higher when compared to the histotype A sample. On average, vascular canals take up between 10% to nearly 20% in histotype B bone tissue (Figs. 9, 10 and 11). The bone tissue is dominated by incipient fibrolamellar bone (Figs. 10, 11, 13, 14J–R). Three subtypes of histotype B can be distinguished (Figs. 10, 11, 13). Some samples show an arrangement of vascular canals in rows (Fig. 10G–I) and, therefore, a stratification of the cortex by changes in bone tissue and vascular canal organization and not primarily a stratification by growth marks, such as annuli or LAGs. Others show no order of vascular canals. The latter can be divided into those samples that show a cortex divided by LAGs and an abrupt change in bone tissue in the outer cortex to an EFS (Fig. 10D–F), and in those that show a stratification of the cortex by diffuse but thick annuli (Fig. 10A–C). Growth rate in histotype B samples decreases generally continuously from the inner to the outer cortex and does not show an alternating pattern of slow and fast growth phases as described for the histotype A sample. Only small humeri, with a length less than 5.0 cm, show histotype B bone tissue.

Histotype B samples do not represent juveniles of histotype A for several reasons, such as differences in bone tissue, vascular density, and distribution of the endosteal bone along the medullary cavity, as well as due to the occurrence of ontogenetically young and old individuals in both histotypes. Furthermore, the initial phase of moderate growth in histotype A samples is not comparable with the highly radially vascularized bone tissue in histotype B samples. Generally, histotype B samples did not show small longitudinal canals, which are numerous in the cortex of histotype A samples (Fig. 9).

#### Growth Mark Count and Asymptotic Growth

In nothosaurs growth marks appear mainly as LAGs, which represent a real growth stop, whereas annuli, which represent a period of slowed down growth, are relatively rare (Figs. 6, 7 and 8). This is contrary to the histotype A sample, where distinct LAGs are rare but annuli stratify most of the cortex (Figs. 6G, J, K, L, 9F, 12). In histotype B subtypes most annuli are completed by a LAG.

In spite of the frequent occurrence of annuli, counting them remains difficult for several reasons. Annuli are often diffuse, which means that they are incompletely developed around the circumference of the cross section. Sometimes they vanish on certain sides of the bone due to the thinning of the cortex or they are not well delimited and vanish in high amounts of lamellar bone. Vis-à-vis, it is not clear if localized aggregations of lamellar bone are the result of an annulus, and if they represent one or several short (seasonal) periods of nearly ceased growth. Furthermore, growth marks of the same sample are highlighted differently in polarized and normal light. Castanet et al. [34] listed several reasons as practical problems in counting growth marks. For example, the diffuse appearance of annuli can be related to the constant environmental conditions and lack of clear seasonality [34]. To standardize growth mark count in the current sample, the number of growth marks given for each specimen (Table S1) refers only to those which are visible in normal light and which can be tracked through nearly the entire cross section. The high number of diffuse annuli throughout the entire cortex makes an annual deposition very unlikely, although the unlikely possibility remains that all of the growth marks observed are truly annual and that Lower Muschelkalk eosauropterygians had a long longevity. Alternatively, these annuli are not annual and contrary to Castanet [34] reflect a strong seasonality, representing several changes in climate during a year. Another observation is that annuli thickness mainly in the nothosaur and histotype A sample does not decrease consistently from the inner to the outer cortex, but it is often irregular. Some samples have very thick annuli already in the inner cortex.

The amount of lamellar bone generally increases towards the outer cortex, but this pattern is repeatedly interrupted by phases of faster growth indicated by poorly organized parallel-fibered bone tissue or even fibrolamellar bone. Thus, it could not be determined for sure if asymptotic growth or full size, respectively, is reached. Only a few samples (e.g., IGWH-17, -20, Wijk09-58) show well developed lamellar zonal bone in the outer cortex, interpreted as an EFS [sensu 44].

#### Medullary Cavity and Endosteal Bone

A Kolmogrov-Smirnov Test documents that the medullary cavity of nothosaur humeri is significantly larger (median  = 65% of total humeral area) than that of pachypleurosaur humeri (median  = 32% of total humeral area), with N = 32 (nothosaurs = 10, pachypleurosaurs = 22) and p<0.01 (see also Table S1, Figs. 7, 11, 12). In *Nothosaurus*, the cavity is often located on the ventropostaxial side, unlike a more dorsopreaxial position in “pachypleurosaurs”. The medullary cavity in femora is nearly central in both groups, except for IGWH-2, which originates from a very young individual (Figs. 7, 13). The very large medullary region in *Nothosaurus* humeri is usually partially filled by a mixture of a primary matrix, primary trabeculae, and diagenetically overprinted secondary matrix (Fig. 7; Table S1). The primary matrix can contain calcified cartilage, distal or proximal to the midshaft. Additionally, vesicles occur in *Nothosaurus* (Fig. 7D–F), which possibly are the product of soft tissue such as adipose. Some *Nothosaurus* samples show small to large calcite crystals in their medullary cavity (Table S1).

In the “pachypleurosaur” sample the medullary cavity is generally smaller, but its size largely depends on the sampling location along the midshaft. The medullary cavity is smallest at midshaft, and its size increases in samples proximal and distal to the midshaft. The “pachypleurosaur” humeri from Winterswijk often have the originally hollow medullary cavities filled with large calcite crystals, whereas in “pachypleurosaur“ humeri from Freyburg only small calcite crystals occur.

The medullary cavities of *Nothosaurus* are sometimes lined by a thin and irregular layer of endosteal bone that seldom surrounds the entire margin (Fig. 7, best visible in samples figured in polarized light). All “pachypleurosaur” samples show endosteal bone along the medullary cavity, except for IGWH-2 (Fig. 12L–N). Development of endosteal bone starts on the postaxial side of the bone. Endosteal bone is present at midshaft but disappears in samples distal or proximal to the midshaft. In pachypleurosaur histotype A, the medullary region is completely filled (Fig. 12B, H, W) or thickly lined by endosteal bone (Fig. 12K, N) (Table S1). In histotype B, the medullary cavity is also lined by endosteal bone, but is never completely or even half filled.

*Nothosaurus* and “pachypleurosaur” samples distal or proximal to the midshaft have the medullary cavity surrounded by a very sharp line and contain calcified cartilage in a primary matrix (Table S1). However, calcified cartilage occurs much closer to the midshaft in *Nothosaurus* than in “pachypleurosaurs.”

### *Nothosaurus* Humeri

Three valid nothosaur taxa were described from the Lower Muschelkalk of the Germanic Basin: *N. marchicus* [1], *N. winterswijkensis* [6], and *N. winkelhorsti* [8]. Only *N. marchicus* is a nearly complete skeleton, including humeri [45].

Three humeri morphotypes (I, II, III) of *Nothosaurus* were described by Bickelmann and Sander [14] from the Lower Muschelkalk. The current sample comprises their morphotype II [14], which is assigned to *N. marchicus/winterswijkensis*, and a new *Nothosaurus* humerus morphotype, which is described below.

#### *N. marchicus/winterswijkensis*

IGWH-25, IGWH-28, and Wijk05-09 can be assigned to morphotype II [14]. The shape of the cross section is triangular-oval, with a smooth convex dorsal side and a straighter ventral side (Fig. 7A–F). The size of the medullary cavity is moderate when compared to morphotype IV (Table S1, Fig. 7). The postaxial and preaxial constrictions of the proximal head are apparent [14]. Unlike morphotype IV, the deltopectoral crest and the latissimus dorsi insertion are not set off by channels in postaxial view. Instead they are divided by a small but distinct ridge running from the facet margin down to a level where the deltopectoral crest and latissimus dorsi insertion start. In relation to the shaft, the proximal end is elongated (Fig. 3G, H). In dorsoventral view, the preaxial proximal edge of the shaft is not prominent. The proximal articulation surface has only a short ventral bulge but a pronounced preaxial bulge (Fig. 3G, H).

#### Histological and Ontogenetic Remarks on *N. marchicus/winterswijkensis*

Bone growth in *N. marchicus/winterswijkensis* shows alternating phases of slow and fast growth (Figs. 7A–F, 8D–F). The inner cortex forms a thick layer of poorly organized parallel-fibered bone tissue, containing woven bone and indicating an initially rapid growth rate. This layer then grades to higher organized parallel-fibered bone tissue consistent with a slower growth phase, which is stratified by two to four growth marks. Attached to this, a second layer of fast growth follows. Finally, the amount of lamellar bone increases again within a highly organized parallel-fibered bone tissue. The outer cortex of older individuals is entirely made of lamellar bone tissue, finally resulting in an EFS. Growth marks are distinct throughout the cortex and easy to distinguish from the surrounding bone tissue.

The three humeri represent a growth series, based on their size, morphology, and histology. The complete left humerus IGWH-25 has a well-developed deltopectoral crest and latissimus dorsi insertion (Fig. 3H). Vascularization is moderate (Figs. 6E, 7A–C). Fibrolamellar bone developed locally in an outer layer of the cortex. The complete right humerus IGWH-28 has a less developed deltopectoral crest and latissimus dorsi insertion (Fig. 3G) when compared to IGWH-25. IGWH-28 shows less lamellar bone and a higher number of osteocytes in its primary cortex (Fig. 7D–F). Longitudinal vascular canals are concentrated on the preaxial and postaxial sides but vascular density is generally low. Funnel-shaped simple canals are numerous (Fig. 8E–G). Wijk05-09 is the proximal end of a left humerus, and the thin section was taken proximal to the midshaft. Vascular density is relatively high, with simple longitudinal canals concentrated in the inner cortex and towards the preaxial side. Postaxially, radial canals dominate the outer cortex. Some primary osteons occur and locally fibrolamellar bone is developed. One annulus has developed in the middle cortex. The ventral cortex contains traces of Sharpey's fibers. The preserved bone tissue suggests a younger ontogenetic stage when compared to the other two specimens.

#### *Nothosaurus sp.* (Morphotype IV)

IGWH-8, IGWH-7, IGWH-14, IGWH-18, and IGWH-30 cannot be assigned to an existing morphotype [sensu 14]. However, they form ontogenetic variations of a new morphotype, the following described as morphotype IV. The most complete specimen showing the features of morphotype IV is IGWH-8 (Figs. 2A–D, 3A, 6C, D, 7G–I, 8K–M). Morphotype IV is characterized by a prominent and protruding edge, seen in dorsoventral view, formed by the very straight preaxial proximal shaft margin. Thus, the proximal end is sharply angled preaxially relative to the shaft. Additionally, the proximal end is shorter in relation to the shaft. At the proximal facet margin, a distinct strangulated bulge has developed ventropostaxially (Fig. 2C). In postaxial view, the deltopectoral crest and the latissimus dorsi insertion are set off by two distinct channels which meet on the proximal shaft (Fig. 2B). The cross section of morphotype IV is more angled when compared to morphotype II (Fig. 7A–L) because the preaxial side of the shaft tapers and is constricted. Morphotype IV can reach lengths twice the size of morphotype II and is generally more massive. The taxonomical assignment of morphotype IV is not yet clear. It could represent an additional species of *Nothosaurus* or a larger sexual morph of *N. marchicus/winterswijkensis*.

#### Histological and Ontogenetic Remarks on Morphotype IV

The growth pattern of morphotype IV differs from that of morphotype II in showing a continuous increase of bone tissue organization and no alternating pattern of slow and fast growth (Figs. 7G–L, 6C, 8K–M). The inner cortex consists of a layer of poorly organized parallel-fibered bone tissue, representing an initial phase of relative fast growth. The growth rate then decreases, and the rest of the primary cortex consists of more organized parallel-fibered bone tissue with an increasing amount of lamellar bone. However, areas of faster growth, indicated by poorly organized parallel-fibered bone, also occur locally in the upper half of the cortex. Annuli are more diffuse when compared to morphotype II, due to a greater proportion of lamellar bone throughout the entire cortex (Fig. 7, 8E, L).

IGWH-30 is the proximal part of a left humerus. It is the largest of this group, and exhibits the most pronounced development of the morphological features described above (Fig. 3B). Postaxially, the proximal head is additionally set off by a dorsoventral channel below the facet margin. No histological sample was taken from this specimen because the midshaft is not preserved. IGWH-8 is a distally incomplete right humerus (Figs. 2A–D, 3A, 6C, D, 7G–I, 8K–M). The position of its deltopectoral crest is indicated by rugosities which extend to the margin of the proximal articulation facet. Vascular density is generally low in this sample, except for the inner cortex. Except for the postaxial bone side this sample shows at the outer cortex a layer of lamellar zonal bone (Figs. 6D, 8K–M). IGWH-18 is a distally incomplete left humerus. The latissimus dorsi insertion is textured by rugosities (Fig. 3C). Channels on the proximal head are also developed. The more distal sample shows no endosteal bone, but a sharp line surrounds the medullary cavity. Vascularization is moderate (Fig. 6B, 7J–L). IGWH-14 is a distally incomplete left humerus with no developed facet margin (Fig. 3D). Morphological features are not distinct and vascularization is moderate. This specimen has an extremely thin postaxial cortex which ventrally contains a high amount of lamellar bone. IGWH-7 is a proximally and distally incomplete left humerus (Figs. 3E, 6A). Its primary cortex is moderately vascularized.

#### Additional *Nothosaurus* Humeri

IGWH-3 is a complete left humerus and represents the smallest sampled nothosaur humerus (Figs. 3F, 7M–O). Its preaxial margin is straight, the shaft is not constricted, and the distal end is not broadened. An entepicondylar foramen is missing. Unlike the midshaft sample, the sample distal to the midshaft shows no endosteal bone. A thick layer of poorly organized, but well vascularized, parallel-fibered bone tissue forms the entire inner half of the cortex. The outer cortex consists of highly organized parallel-fibered bone tissue and also contains a high amount of lamellar bone (Fig. 7N). Although morphology and size point to a young individual, bone tissue and growth mark count (Table S1) contradict this. However, the continuous increase in bone tissue organization from the inner to the outer cortex resembles a growth pattern similar to morphotype IV. IGWH-4 is the distal end of a right humerus from a very large individual with a very simple distal morphology (Fig. 3I). The distal end is not broadened when compared to the shaft. Vascular density is low. The outer cortex mainly consists of lamellar zonal bone tissue. However, the sample is not easily comparable to other specimens, due to its sampling location distal to the midshaft. IGWH-17 is the poorly preserved distal end of a left humerus (Figs. 3J, 8H–J), and it was sectioned distal to the midshaft. The vascularization density is low. Growth pattern is most likely alternating, like in morphotype IV samples. In any case, IGWH-17 represents the oldest individual in the current sample set, because its outer half of cortex consists of nearly pure lamellar zonal bone tissue, clearly representing an EFS.

### “Pachypleurosaur” Humeri

Pachypleurosaurs are known from three valid taxa in the Lower to Middle Muschelkalk of the Germanic Basin: *Dactylosaurus gracilis*, *Anarosaurus pumilio* and *A. heterodontus* [1], [7]. None of the humeri sampled here matches the humeral morphology of *Dactylosaurus* or *A. pumilio*. Unfortunately, only one skeleton of a juvenile *A. heterodontus* contains a humerus, which makes it quite difficult to diagnostically describe and compare its morphology [7]. *A. heterodontus* is the only pachypleurosaur so far described from Winterswijk and is the most common fossil there [7]. No postcranial elements are known from *Cymatosaurus*, the basal member of the Pistosauroidea from any locality [1].

Humeri classified here as the “pachypleurosaur” type exhibit a very diverse morphology, and combining them into morphological groups is problematic. However, histology of these humeri allows an allocation of at least two major histotypes as described above. Additionally, the large humeri showing histotype A can be combined on the basis of their distal morphology into two morphological groups. Histotype B can be distinguished into further histological subtypes but not into morphological groups, because none of these smaller humeri looks alike (Fig. 5). It must be pointed out that both proposed histotypes may comprise several taxa or sexual morphs.

### “Pachypleurosaur” Humeri Showing Histotype A Bone Tissue

#### Morphotype I

Humeri IGWH-29, NMNLH St 449487, IGWH-19, and IGWH-20 share a similar distal morphology that entails a proximally shifted entepicondyle (Fig. 4B, C). The capitellum is located on the preaxial half of the bone. It is postaxially followed by a distinct ectepicondylar groove and a proximally located small ectepicondyle. IGWH-29 is a complete right humerus with a damaged postaxial distal margin (Fig. 4D, 6L). Partially, primary osteons have formed. Vascularization is only moderate when compared to all other histotype A samples (Figs. 6G–L, 10). The initial phase of moderate growth forms two thirds of the sample's entire cortex and is followed by a phase of slower growth which contains large amounts of lamellar bone. NMNHL RGM 449487 is a complete left humerus (Fig. 4C, 6I). Several vascular canals reach the outer cortex and are connected to the bone surface. The vascular density is distinctly higher when compared to IGWH-29 (Fig. 6G–L). Partially, primary osteons have formed. Here, the initial moderate growth phase terminates in a thick annulus, which is not visible in normal light (Fig. 12J–L). Preaxially, the outer cortex consists of lamellar bone tissue, whereas postaxially the beginning of a fast growth phase is indicated by a relatively poorly organized parallel-fibered bone tissue and an increasing number of radial canals. The sample shows a cavity left by a foramen in the middle of the postaxial cortex. IGWH-19 is a distally incomplete left humerus (Figs. 2E–H, 4A, 6K, 12G–I, 15). However, based on additional material in the IGWH collection and according to Rieppel's description [43], the distal morphology of this humerus matched the above described characters, including a proximally shifted entepicondyle. The bone surface of IGWH-19 is rugose at the latissimus dorsi insertion and the deltopectoral area. The proximal end is elongated in relation to the shaft and appears generally angled and edged. The postaxial proximal side is marked by two deep channels running dorsally and ventrally down from the proximal head (Fig. 2F). The latissimus dorsi insertion lacks a distinct ridge, but instead forms a depression, unlike all other studied humeri (Fig. 2E). The preaxial proximal side is asymmetrically divided by the sharp preaxial margin into a smaller steep ventral part but with a less angled dorsal surface when compared to *Nothosaurus*. However, the preaxial margin has a prominent rhombic indentation, indicating the attachment of the pectoralis muscle. The shaft is clearly constricted and the distal end broadens to a fan-shape. The entepicondylar foramen is large and is clearly at a distance from the distal margin. The medullary cavity has shifted to the dorsal side in midline axis, resulting in a very thick ventral cortex. In IGWH- 19, as well as in IGWH-20, several alternating fast and slow growth phases follow the initial moderate growth phase (Fig. 15), containing distinct layers of fibrolamellar bone (Table S1). IGWH-20 is the distal end of a right humerus (Figs. 4B, 6J, 9B, 14G–L). The broad angled area between the entepicondyle and capitellum is concave and marked by rugosities. The specimen shows two separated layers of avascular lamellar bone tissue in its outer cortex, most likely representing an EFS (Fig. 14G–L).

#### Morphotype II

Humeri NMNLH St 445912, IGWH-1, and IGWH -26/27 are also grouped together because of a similar distal morphology (Fig. 4E–G). They show steeply angled preaxial and postaxial margins in dorsoventral view, due to a proximal shift of ectepicondyle and entepicondyle. The capitellum is prominent, sometimes distally protruding, and located nearly centrally. The proximal end is twisted (mainly in postaxial view) when compared to the distal end. NMNHL ST 445912 is a distally incomplete left humerus (Fig. 4E). The proximal end is laterally constricted and very flat. No histological sample was taken from this bone. IGWH-1 is a complete right humerus (Figs. 4F, 6H, 12D–F, 14A–F). The distal end is damaged but the position of the capitellum is clear. An entepicondylar foramen was not identified. The cortex is atypically well stratified by a high number of annuli (Table S1) which, however, are not visible in normal light. IGWH-26/27 is a complete left humerus (Fig. 4G, 6G). An entepicondylar foramen is not visible, possibly due to damage in this area. Proximal to the midshaft, remains of primary trabeculae occur at the margin of the medullary cavity. Both samples show alternating growth phases and layers of fibrolamellar bone (Table S1).

#### Additional “Pachypleurosaur” Humeri Showing Histotype A Bone Tissue

IGWH-11 is a complete right humerus (Figs. 4H, 12P–R) with a well textured proximal head. A distinct ectepicondylar groove is not developed, but the entepicondyle is located proximally when compared to the rest of the distal end. The entepicondylar foramen is small and set at a distance from the distal margin. The medullary cavity is very large for a “pachypleurosaur.” The sample distal to midshaft shows a primary trabecular structure in its medullary cavity. Starting at this trabecular system, large cavities have eroded the cortex. They are lined by endosteal bone and diagenetically filled by crystalline growth. The vascularization is moderate and dorsally dominated by radial canals. Primary osteons have only partially developed, which is similar in IGWH-12. In both samples the cortex represents the initial phase of moderate growth of histotype A. IGWH-12 is a distally incomplete right humerus (Figs. 4I, 12S–U). The proximal end is not as rectangular as but is longer than IGWH-11. The bone surface is, except for the latissimus dorsi insertion, not textured. Although the sample was taken at midshaft, the medullary cavity in this specimen is also very large. Ventrally, vascularization is greatest and dominated by radial canals. The inner cortex shows a fractional amount of fibrolamellar bone. IGWH-16 is an incomplete right humerus with no distinct morphological features (Figs. 4J, 9A, 12V–X). The vascular density is high and dominated by radial canals. Primary osteons have partially started to form. The inner and middle cortex represents the initial phase of moderate growth with fibrolamellar bone already intercalated. IGWH-10 is a proximally incomplete left humerus with a simple and flat morphology (Figs. 4K, 12M–O). The entepicondylar foramen is large and located close to the distal margin. The larger medullary cavity in the thin section distal to the midshaft is surrounded by a thick layer of endosteal bone, as well as by a sharp line (Fig. 12M–O). The cortex is highly vascularized and contains longitudinal and radial canals with partially developed primary osteons. Bone tissue represents the initial moderate growth phase of histotype A. IGWH-6 is the proximal end of a right humerus (Fig. 4N). Its bone tissue, as well as that of IGWH-22, shows several alternating growth phases intercalated with layers of fibrolamellar bone. IGWH-22 is a distally incomplete left humerus (Fig. 4M). Four samples from the proximal shaft to the midshaft were taken. Differences between these four samples mainly concern the size of the medullary cavity, which increases distally and proximally. In the most proximal sample, the medullary cavity is surrounded by a sharp line. Additionally, the medullary cavity is no longer lined by endosteal bone but is completely filled by primary trabeculae and calcified cartilage. Traces of Sharpey's fibers are preserved on the postaxial ventral side of the medullary cavity. In the thin sections proximal and distal to the midshaft, the growth record is incomplete. IGWH-15 is the distal end of a left humerus with a damaged postaxial margin (Fig. 4L). The shaft is constricted. The distal morphology is typical for the “pachypleurosaur” type and the prominent ectepicondyle is separated by a distinct ectepicondylar groove from the capitellum. Due to the sampling location distal to the midshaft, the large medullary cavity is surrounded by a sharp line and not lined by endosteal bone. Because of the distal sampling location, the growth record is incomplete. Bone tissue is dominated by longitudinal primary canals. IGWH-31 is the proximal end of a left humerus (Fig. 4O) with distinct morphological features. The postaxial proximal end is flattened. No histological sample was taken from this specimen because the midshaft region is not preserved.

#### Morphological and Ontogenetic Remarks on Humeri Showing Histotype A Bone Tissue

Humeri of “pachypleurosaur” morphotype I and II show differences in proximal morphology and size, and several species or sexual morphs may be included. However, their histology and that of additional humeri of the “pachypleurosaur” type can be regarded as consistent, indicating a taxonomic group. In the following, the ontogenetic stage is addressed for each specimen on the basis of histology.

In histotype A, growth marks occur mainly in the form of annuli that are not visible in normal light and which are often difficult to assess as mentioned before. Furthermore, the infilling of the medullary cavity by endosteal bone, which is normally an indication that endochondral ossification has finished [24], reveals in the histotype A samples no clear signal for adulthood because the infilling by endosteal bone is already completed in young individuals (e.g., IGWH-5, IGWH-16).

Because of the difficulties in counting growth marks, growth classes are defined for histotype A samples on the basis of the main changes in growth (Table S1; Fig. 15). However, differences in the respective growth of each individual occur, and are normal, because growth strongly depends on external and internal factors [34] and varies in each individual. Size class I includes specimens showing only the initial phase of moderate growth (IGWH-10, IGWH-11, IGWH-12, IGWH-16; Fig. 12M–X) which is interpreted as a juvenile stage of individuals in their first or second year. Size class II contains those samples which show the initial moderate growth phase with a hereinafter distinct change in bone tissue and vascular organization towards a faster growth phase. This change is often accompanied by one or two closely spaced growth marks (IGWH-1, IGWH-26/27, IGWH-29, NMNHL RGM 449487; Figs. 12A–F, J–L, 15). Size class III comprises a phase of slow growth which follows the initial moderate and first fast growth phase. The three main changes in growth are well separated by three distinct growth marks (IGWH-6, IGWH-19; Fig. 12G–H). Specimens of size class IV are characterized by the presence of lamellar zonal bone tissue in the outer cortex representing an EFS (IGWH-20, IGWH-22; Fig. 14G–L).

During the different growth phases, two growth marks are most distinct: the one which terminates the initial moderate growth phase and the one which terminates the first phase of fast growth. It is likely that one of these growth marks is accompanied by the onset of sexual maturity, but which one cannot be resolved.

### “Pachypleurosaur” Humeri Showing Histotype B Bone Tissue

#### Internal Cortex is Continuously Vascularized Until Bone Tissue Changes to Lamellar Zonal Bone in the Outer Cortex

Wijk09-58 is a complete right humerus with a complex morphology (Figs. 5B, 10D–F, 11A–C, 14M–R). The proximal end is short and rectangular in dorsoventral view. The postaxial proximal end has a bulge below the head, and the specimen appears massive. Postaxially, the vascular canals in the middle cortex are larger than in the inner and outer cortex. The outer cortex shows two distinct layers of lamellar zonal bone, representing an EFS (Fig. 10D–F). Wijk08-219 is a complete right humerus entirely textured by striations (Figs. 5E, 11D–F). The bone is dorsoventrally flat and has a simple morphology. The proximal end appears short, but it is neither rectangular nor angled like in others (e.g., Wijk08-183, Fig. 5). The outermost cortex shows an increasing amount of lamellar bone. Wijk06-238 is the proximal end of a left humerus (Fig. 11G–I). Postaxially, the medullary cavity shows an irregular layer of endosteal bone with numerous and thick osteocytes inside. In the sample proximal to the midshaft, a sharp line surrounds the medullary cavity. The outer cortex shows only locally lamellar bone tissue.

#### Cortex Stratified by Annuli

Wijk07-50 is a complete right humerus (Figs. 5C, 10A–C, 11J–L). The specimen shows a long proximal end with an oblong head and an overall slender appearance. In the upper third of the cortex, lamellar zonal bone has developed. However, the lamellar zonal bone is followed by a layer of poorly organized parallel-fibered bone tissue, which is finally delimited again by another layer of lamellar zonal bone in the outermost cortex. Wijk07-137 is a complete right humerus with simple morphology (Figs. 5F, 11M–O). Wijk09-543 is a complete left humerus (Figs. 5D, 11P–R). The nearly rectangular proximal end is very prominent when compared to the small constricted shaft and the flat broadened distal end. Simple canals dominate, although primary osteons have partially formed. Wijk09-472 is a sample from a complete left humerus (Figs. 5C, 11S–U). The proximal end is dorsoventrally compressed, and the outer cortex shows a high amount of lamellar bone.

#### Vascular Canals Are Arranged in Rows

Wijk08-183 is a complete left humerus (Figs. 5A, 10G–I, 11Y–a). Its morphology is somewhat atypical because the proximal end is flat in pre-postaxial direction but massive. An indentation on the preaxial ventral side is missing. The distal morphology is relatively simple, with a proximally shifted ectepicondyle and a preaxially located capitellum. Postaxially, where the cortex is thickest, no endosteal bone has developed, and large primary radial osteons are arranged within layers of fibrolamellar bone. Preaxially, vascular density is lower. Wijk07-70 is the proximal end of a left humerus. The radial canals are arranged in rows (Fig. 11b–d). The outermost cortex consists of lamellar zonal bone. NME48000085c originates from a long bone fragment. It is presumed to belong to a humerus because of the decentralized medullary cavity and its oval cross section (Fig. 11V–X). The radial canals are roughly arranged in rows.

#### Morphological and Ontogenetic Remarks on Humeri Showing Histotype B Bone Tissue

The morphology of humeri showing histotype B varies so much that no two are alike. Their only similarity is their size range, because no humerus is larger than 5.0 cm. However, their morphology and size clearly differ from those humeri showing histotype A. Histotype B samples can be divided into histological subtypes as described above, but it is not clear if these differences are taxonomically relevant, simply reflect variations in individual's growth and life history, or represent sexual morphs.

Ontogenetic stages can be addressed for histotype B specimens. Growth marks in histotype B samples are more distinct when compared to histotype A samples, because annuli are often accompanied by a LAG. Overall growth pattern in all three subtypes of histotype B is characterized by an initial phase of very rapid growth. After this initial phase, fast growth continued until the bone tissue finally changed to lamellar zonal bone in the outer cortex. Specimens showing no layer of lamellar zonal bone or an EFS in the outer cortex can be regarded as “juveniles,” or at least as individuals not fully grown, whereas specimens which show a thick layer of lamellar zonal bone in the outer cortex can be interpreted as adults or nearly fully grown individuals. However, in all three subtypes, lamellar zonal bone is repeatedly interrupted by layers of faster growth, and asymptotic growth cannot be determined for sure.

In Wijk06-238 and NME48000085c no growth marks are identified. They thus represent the youngest specimens. Wijk09-543 and Wijk08-219 may have been in their second or third year (Table S1), and Wijk07-137 and Wijk08-183 in their fifth (Table S1). Wijk07-70 is difficult to interpret because its inner cortex shows no growth marks, but lamellar zonal bone is already deposited in the outermost cortex, divided by two LAGs, suggesting an age of at least three years. In Wijk09-472 and Wijk07-50, the cortex is stratified by four and five thick annuli, suggesting an age of five and six years, respectively. Wijk09-58 clearly represents the oldest histotype B sample due to a thick layer of lamellar zonal bone ( = EFS) in the outer cortex, with an age of at least seven years.

### Placodontia

IGWH-9 is a complete left humerus. The simple morphology and roughly crescent-shaped form resembles that of placodonts. The shaft is not constricted as in “pachypleurosaurs,” nor is its preaxial margin as straight as in *Nothosaurus*. The proximal shaft carries a foramen on the postaxial half of the ventral side. The distal end is very broad and massive. The entepicondylar foramen is only dorsally present, and it is located close to the postaxial distal margin. The vascular canals take up as much space as the bone tissue (Fig. 13A, B). Vascular density is even higher than in histotype B and is much more irregular. The vascularization is dominated by very large longitudinal canals, representing more holes than canals (see also IGWH-23 in Fig. 13C–E). Radial canals can also occur. Vascular canals are locally arranged in rows. Primary osteons have partially formed. High vascularization in the middle cortex is interrupted by two layers of poorly vascularized parallel-fibered bone tissue.

### Sauropterygia indet

IGWH-13 is a distally incomplete and damaged left humerus. It is very straight, without a distinct angle between the shaft and the proximal end. It is not clear if the shaft was constricted or not. The proximal shaft carries a foramen on the center of the postaxial side. The vascularization pattern is generally radial, but a large number of thick and large longitudinal canals occur, too. The vascular canals are arranged in layers, at least in the inner cortex. Primary osteons are numerous. The inner cortex consists of parallel-fibered bone tissue with intercalated layers of fibrolamellar bone. Bone histology and growth pattern is similar to histotype A, except for the higher vascular density and the prominent large longitudinal to irregular canals which are more similar to those of placodonts (IGWH-9, IGWH-23; [23]).

### General Femur Morphology of Eosauropterygia

**Figure 16:**
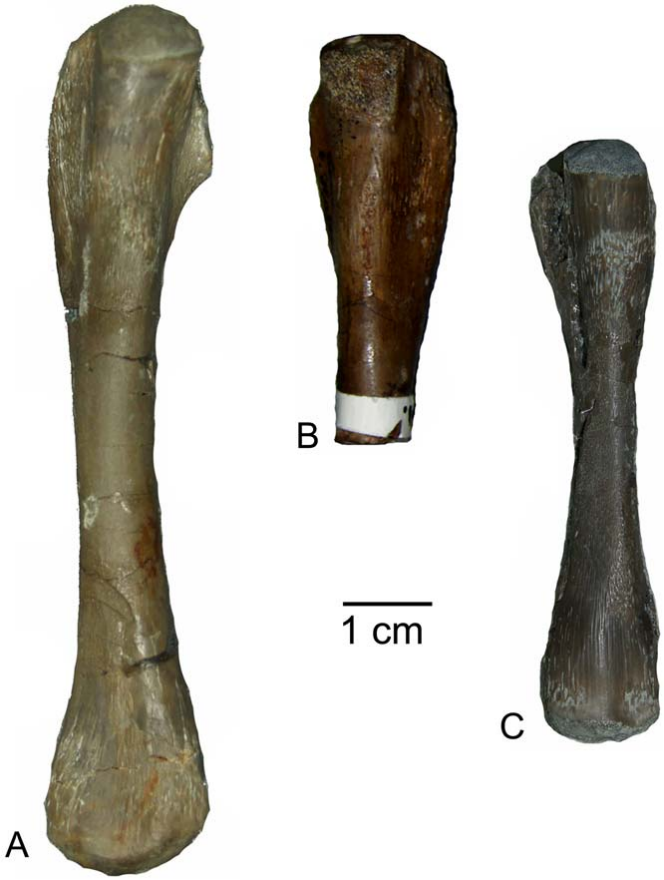
Femora of Eosauropterygia. A) Left femur Wijk05-10 which is on the basis of histology assigned to *Nothosaurus*. B) Right femur IGWH-24 which is on the basis of histology assigned to *Cymatosaurus* (histotype A). C) Right femur Wijk08-150 which is on the basis of histology assigned to *Anarosaurus heterodontus* (histotype B). A) and B) were figured before sampling.

Eosauropterygian femora are long and slender, and the femora of different taxa are not morphologically distinguishable (Fig. 16). Their proximal articular surface is triangular, with a thick and round protruding muscle insertion. Ventrally, smaller postaxial and preaxial ridges occur. In dorsal view, the postaxial proximal ridge is located distally when compared to the preaxial and dorsal ridge. The postaxial side carries proximally a broader and more curved flange (trochanter) than the preaxial side. Postaxial and preaxial side can carry elongated foramina. A small but distinct crest lies below the prominent dorsal muscle insertion of the proximal articulation surface. The shaft is straight and constricted, with a rounded triangular cross section. The dorsal shaft margin is straight, but the ventral shaft margin is slightly concave. In dorsal view the postaxial margin is straight, and the preaxial margin is slightly concave. The flat distal end broadens only slightly. In some specimen the distal ends point somewhat to the preaxial side. The equally sized condyli are located ventrally.

### General Femur Histology of Eosauropterygia

Bone histology of femora is similar when compared to the humeri, and femora can be assigned on the basis of their histology to placodonts and nothosaurs as well as to the “pachypleurosaur” histotypes. Cross sections of femora are easy to distinguish from humeral cross sections (Figs. 7, 12) because of their straight or even concave ventral side. Femora exhibiting histotype A have the margin of the nearly central medullary cavities lined by endosteal bone, but none of the samples shows complete infilling (Figs. 9E, F, 12F–Q). Furthermore, histotype A femora have an alternating growth pattern similar to that observed in the humeri (Fig. 9E, F). The initial moderate growth phase, dominated by small to large longitudinal canals, is followed by a phase of slow growth, indicated by lamellar zonal bone tissue. Only then is a phase of faster growth developed. Generally, the cortex of femora contains a greater amount of lamellar bone and is less vascularized when compared to the cortices of humeri. Femora exhibiting histotype B show all three subtypes observed in the humeral samples (Fig. 13R–Z).

### *Nothosaurus* sp

Wijk05-10 is a complete, large left femur (Fig. 16A). The dorsal proximal crest and the textured intertrochanter fossa are well developed. The postaxial proximally protruding muscle insertion is damaged, but it is unclear if this is pathologic or if it happened postmortem (e.g., a drilling hole of an invertebrate) or during fossilization. Although the specimen is cut at midshaft, the medullary region is very large (Fig. 7P–R). The proper medullary cavity occupies only 16.5% of the radius, but the entire medullary region occupies 60% in the pre-postaxial direction. The vascularization is moderate and dominated by small radial to longitudinal canals. Partially, primary osteons have formed and the middle cortex contains a thin layer of fibrolamellar bone (Fig. 8A–C). On the basis of its bone tissue Wijk05-10 surely belonged to an adult, but asymptotic growth was not reached.

### Femora Showing Histotype A Bone Tissue

IGWH-21 is a distally incomplete left femur. A facet margin is not identified, but this is maybe due to poor preservation. The intertrochanteric fossa is covered by striations and grooves. Because of the high number of LAGs and four alternating growth phases (Table S1), IGWH-21 belongs to an adult, nearly fully grown individual. IGWH-24 is a distally incomplete right femur (Fig. 16B). The dorsal crest and the intertrochanteric fossa are distinct. Bone tissue and alternating growth phases are similar to IGWH-21, except for a higher vascular density and a much thicker innermost growth phase with large longitudinal canals (Figs. 8E, F, 13I–K). A phase of fast growth is missing in IGWH-24, most likely due to an ontogenetic younger stage. NME48000074 originates from a long bone fragment but shows the typical femoral cross section. The outer cortex shows alternating layers of lamellar zonal bone tissue (Fig. 13F–H). Preaxially, the cavity of a large foramen is preserved in the middle of the cortex. Bone tissue in NME48000074 is very similar to humerus NMNHL RGM 449487 except for the clear stratification of the femur by growth marks (Table S1).

NME48000085a originates from a long bone fragment, and its cross section and the centrally located medullary cavity match that of a femur. The vascularization density is relatively high. In the outer third of the cortex is the cavity of a large foramen. IGWH-5 is a distally incomplete left femur. A facet margin is not developed, but the entire articular surface and the intertrochanteric fossa are covered by striations and grooves. A foramen is obvious on the preaxial side of the proximal end. No crest is visible below the prominent dorsal muscle insertion of the proximal articulation surface. Although sampling location was at midshaft, the medullary cavity is surrounded by a sharp line (Fig. 13O–Q). The cortex is dominated by longitudinal canals representing the initial phase of moderate growth which is subsequently followed by lamellar zonal bone tissue. However, because of what is known from the growth in IGWH-21 and IGWH-24, IGWH-5 belonged not to an old individual, as the lamellar zonal bone tissue may predict, but to a juvenile.

IGWH-2 is a complete left femur from a juvenile individual. The distal end is textured by striations, and the condyli are not well separated. A facet margin is not developed, but the entire articulation surface and the intertrochanter fossa is covered by striations and grooves. Neither a postaxial foramen nor a crest below the prominent dorsal muscle insertion of the proximal articulation surface is visible. The medullary cavity is located close to the ventral margin. Periosteal growth has mainly extended to the dorsal side. The medullary cavity of IGWH-2 is also surrounded by a sharp line (Fig. 13L-N). Although changes in color imitate cyclic growth, no real growth marks are deposited. The bone tissue is identical to that described for the innermost cortex of IGWH-21 and IGWH-24. Therefore, IGWH-2 clearly represents a juvenile, and the youngest in the current femur sample, possibly in the entire study.

### Femora Showing Histotype B Bone Tissue

#### Internal Cortex Is Continuously Vascularized until Bone Tissue Changes to Lamellar Zonal Bone in the Outer Cortex

Wijk08-150 is a complete right femur (Fig. 16C). A dorsal crest is only indicated by striations, as is the small intertrochanteric fossa. The postaxial flange is damaged by a crack. The flat distal end is textured by long striations. The bone tissue and growth pattern are similar to that of humerus Wijk09-58. The outer cortex consists of lamellar zonal bone, but the outermost cortex shows poorly organized parallel-fibered bone tissue, again (Fig. 13X–Z). Wijk08-150 belonged to an individual which had reached adulthood.

Wijk06-14 is the proximal half of a poorly preserved right femur. The midshaft region is damaged and only half preserved. Bone tissue changes to poorly vascularized lamellar zonal bone in the outer cortex. Wijk06-14 was most likely younger than Wijk08-150, based on a lower number of growth marks and the absence of lamellar zonal bone in the former (Table S1). Wijk06-84 is a compressed femur with collapsed proximal and distal ends. The outer cortex is separated by one LAG and shows a high amount of lamellar bone, but no EFS. Therefore, Wijk06-84 belonged to a young individual. Wijk07-11 is similarly compressed like Wijk06-84. This individual was very young, based on its high vascular density and the lack of growth marks.

#### Inner Cortex Stratified by Annuli

Wijk07-03 is a complete but laterally compressed left femur. The postaxial proximal muscle insertion forms a distally shifted and descending long ridge. The cross section is oval with a concave ventral side. The vascular canals are aligned, and the bone tissue and growth pattern are similar to that of humerus Wijk07-50. The outer cortex consists of lamellar bone tissue and contains a thick layer of lamellar zonal bone tissue. Wijk07-03 belongs to an adult but was not as old as Wijk08-150. Wijk06-102 is a laterally compressed proximal half of a femur. The vascularization is low. Wijk06-102 was much older than Wijk07-03, based on the lower vascular density, the greater number of annuli, and the general high proportion of lamellar bone throughout the entire cortex (Fig. 13U–W). Wijk06-86 is the proximal half of a left femur. The vascularization is dense, but the cortex also contains a high amount of lamellar bone. This individual was younger than Wijk07-03 and Wijk06-102 because of the higher vascular density and the lower number of annuli. NME48000075 originates from a long bone fragment which most likely belonged to a femur. The inner third of the bone tissue consists of two thick annuli visible in normal light, making this sample somewhat atypical. Wijk09-636 originates from a long bone fragment which most likely belonged to a femur. In the inner cortex, vascularization is dense, otherwise it is only moderate to low. The outer cortex consists of lamellar bone tissue.

#### Vascular Canals Arranged in Rows

WijkHO-A568 is a proximally incomplete femur. The radial canals are arranged in rows (Fig. 13R–T) similar to the condition seen in humerus Wijk08-183.

### Placodontia

IGWH-23 is the incomplete proximal end of a large right femur. The shaft is straight, with a nearly rectangular cross section which is ventrally concave and dorsally carries a smooth peak. The medullary cavity is located on the dorsal preaxial margin and is not separated well from the cortex (Fig. 13C–E). Bone histology is very characteristic and reminiscent of IGWH-9. The vascular canals take up as much space as the bone tissue, but are not dominated by a specific pattern, shape or arrangement. Primary osteons are numerous and fibrolamellar bone has developed. Towards the outer cortex, the amount of lamellar bone increases.

### Comparison

As mentioned above, little research has focused on sauropterygian bone histology. Thus, little is known, except that this group exhibits a broad variety of bone tissues and special adaptations of bone to aquatic life. Most of the previously described material is not comparable with the current sample because other bones of the skeleton were sampled or sampling locations differ.

Sander [22] studied only polished sections, and did not describe bone tissue or detailed histological features of the pachypleurosaur *Neusticosaurus*. However, the neusticosaur sample from Sander [22] was processed into thin sections for the current study. Based on this new information, the dominant bone tissue in *Neusticosaurus* is highly organized parallel-fibered bone containing a high amount of lamellar bone. It can be summarized as LZB type. The vascular density in midshaft samples is low and dominated by simple longitudinal canals. Thus, vascularization patterns differ from the high density, radial patterns observed in histotypes A and B. Furthermore, no fibrolamellar bone was observed in any of the *Neusticosaurus* samples. Osteocytes in *Neusticosaurus* are smaller and their number is less numerous when compared to histotypes A and B. The entire cortex of neusticosaurs is regularly stratified by LAGs, as was previously described [22]. In the current “pachypleurosaur” sample growth marks appear often only as annuli, or annuli are accompanied by LAGs. The most distinctive histological features of *Neusticosaurus* are the large size of the medullary cavity and the presence of calcified cartilage in the medullary cavity all along the shaft region. Bone tissue, cyclicality of growth, and the incomplete endochondral ossification of *Neusticosaurus* largely resemble the histological features observed in Lower Muschelkalk *Nothosaurus* and differ completely from the “pachypleurosaur” sample described in the current paper.

Described *Nothosaurus* long bones from the Lower Muschelkalk [17] most likely did not belong to a nothosaur because they resemble the histology of histotype A, including a small medullary cavity surrounded by thick endosteal bone and the presence of numerous radial canals.

Krahl et al. [26] focused on Upper Muschelkalk *Nothosaurus* and described a typical reptilian LZB type for these taxa, indicating slow cyclical growth. Although bone tissue of Lower Muschelkalk *Nothosaurus* indicates a slower growth rate when compared to the “pachypleurosaurs” sample, *Nothosaurus* growth in the current sample was nevertheless fast at times, contrary to the observations in Krahl [26]. Krahl et al. [26] also studied pistosaur humeri and femora and found that “the femur of *Pistosaurus* is composed of LZB type”, but its humerus “consists of FLB type” [26]. This is contrary to the observations in the current study, where humeri and femora exhibit a similar histology. However, the data of [26] are not yet published in detail.

Wiffen et al. [25] described the histology of Cretaceous *Plesiosaurus* humeri as a “radiating like vascular network” with “intense Haversian remodeling.” Except for the Haversian remodeling, their description of bone tissue from plesiosaurs equals that of the current histotype B sample, leaving interesting consequences for phylogeny which will be discussed below.

Buffrénil and Mazin [23] described the histology of a *Placodus* humerus from the Upper Muschelkalk of Crailsheim. They point out the extensive presence of periosteal “woven-fibered” bone tissue with a very high vascular density, and they inferred a high sustained growth rate for *Placodus* [23]. Their description and figures of placodont bone tissue [23], [24] show similarities to IGWH-9 and IGWH-23. The main differences concern the small size and the sharply from the cortical bone demarcated medullary cavity in their sample [23] relative to the one described here.

## Discussion

### *Nothosaurus* sp

#### Taxonomic Implications

The current sample comprises two *Nothosaurus* humeri morphotypes: morphotype II [sensu 14], which is assigned to *N. marchicus/winterswijkensis*, and a newly described morphotype IV. Morphotype IV represents an additional nothosaur taxon or a larger sexual morph of *N. marchicus/winterswijkensis.* Morphotype II occurs in Freyburg and Winterswijk, but morphotype IV is so far restricted to Freyburg. The existence of now four described nothosaur morphotypes from the Lower Muschelkalk of the Germanic Basin and possibly several more yet undescribed ones [46], [47] (personal observation in the collections of MFN, BGR, IGWH, NME, Table 1), increases the taxonomic diversity of *Nothosaurus* beyond that known from skull morphology.

**Table 1:** List of Institutional Abbreviations.

BGR	Federal Institute for Geosciences and Natural Resources, Berlin, Germany
IGWH	Institute of Geosciences of the Martin-Luther-University Halle-Wittenberg, Germany
IPB	Steinmann-Institute of Geology, Mineralogy und Palaeontology, University of Bonn, Germany
GPLS	Geological-palaeontological collection of the University of Leipzig, Germany
MFN	Museum für Naturkunde, Leibniz Institute for Research on Evolution and Biodiversity at the Humboldt University Berlin, Germany
NMNHL	National Museum of Natural History (Naturalis), Leiden, The Netherlands(including samples labelled with “Wijk”)
NME	TwentseWelle, Enschede, The Netherlands
PIMUZ	Palaeontological Institute and museum of the University of Zurich, Switzerland

#### Long Bone Histology and Growth Pattern of *Nothosaurus*

Lower Muschelkalk *Nothosaurus* grew with cyclically interrupted, parallel-fibered bone tissue which finally graded into an EFS, and asymptotic growth was eventually reached. Only rare areas of localized fibrolamellar bone developed in *Nothosaurus*. Vascular density is moderate or low and simple longitudinal canals dominate. Osteocytes are generally smaller and less numerous when compared to histotypes A and B, but are nevertheless numerous (Figs. 6D, 14J). Thus, bone histology reveals that *Nothosaurus* grew with somewhat higher growth rates than expected, and occasionally even so fast that fibrolamellar bone developed, but bone type can nevertheless be summarized as LZB type [33]. In comparison to histotypes A and B, *Nothosaurus* has more derived humerus morphology but more plesiomorphic bone histology.

The overall growth pattern of *Nothosaurus* is typical for reptiles (e.g., [21], [48]–[50]). However, differences between the growth pattern of *Nothosaurus* morphotypes II and IV occur and therefore support the assignment to different taxa or to different sexual morphs. Whereas morphotype II shows alternating phases of fast and slow growth, bone tissue organization increases continuously in morphotype IV. However, growth mark count in humeri of adult individuals in both morphotypes is similar, with a maximum of six growth marks (Table S1).

#### Unique Histological Features of *Nothosaurus*

The filling and size of the medullary region of Lower Muschelkalk *Nothosaurus* clearly differ in comparison to histotypes A and B. Differences concern the size of the medullary cavity and the possible occurrence of soft tissue (Table S1). They can be interpreted as supplementary adaptations of *Nothosaurus* to the marine habitat, which are absent in Lower Muschelkalk “pachypleurosaurs”. Thus, nothosaurs can be interpreted to be more adapted to the marine life and presumably lived farther offshore, which is consistent with the fossil record [1], [7]. Furthermore, because of the wider distribution of calcified cartilage along the midshaft, *Nothosaurus* humeri are paedomorphic when compared to Lower Muschelkalk “pachypleurosaurs” and reveal a different, more incomplete ossification pattern. *Nothosaurus* shows only a thin, irregular lining of the medullary cavity by endosteal bone and thus generally maintains incomplete endochondral bone formation. This observation is related to general differences in growth and ossification between both groups, but it is not yet understood. Most interestingly, bone tissue and growth pattern, as well as medullary cavity filling, and distribution of calcified cartilage along the midshaft, are similar between Lower Muschelkalk *Nothosaurus* and the late pachypleurosaur *Neusticosaurus*.

### “Pachypleurosaur” Sample

#### Taxonomic Implications

Whereas the *Nothosaurus* humeri are consistent in morphology and represent clear growth series concerning size, morphology, and histology, the “pachypleurosaur” sample is difficult to interpret. Humeri of the “pachypleurosaur” type are not easy to classify in morphological groups because even the two “pachypleurosaur” morphotypes described here, each show differences at least at their proximal ends (Fig. 4A–G). Furthermore, the smaller humeri from Winterswijk cannot be combined into any morphotype, and no two humeri are alike (Fig. 5). As mentioned before, it is not clear if morphological differences are related to intraspecific variation (including sexual dimorphism and ontogeny), or if they represent taxonomic differences. In any case, variability of humeri of the “pachypleurosaur” type is much greater when compared to the “nothosaur” type and points to a higher taxonomical diversity of Lower to Middle Muschelkalk eosauropterygians within the Germanic Basin than known today. However, this can only be clarified by diagnostic finds from the fossil record.

Until proven otherwise, “pachypleurosaur” morphotype I and II are assumed to belong to the same taxon and possibly represent sexual morphs, or alternatively they represent two very closely related taxa. This is because they share at least some morphological similarities, fit into the same size class, form a growth series, and show a uniform histology and growth pattern (Fig. 15). Rieppel [43], [51] proposed that humeri similar to “pachypleurosaur” morphotype I (IGWH-19/IGWH-20) and “pachypleurosaur” morphotype II (NMNHL ST 445912), possibly belong to *Cymatosaurus*. However, this has not been verified because *Cymatosaurus* is only known from isolated skulls (summarized in [1]). The assignment of postcranial material of “*Proneusticosaurus*” [52] to *Cymatosaurus*, as suggested by several authors, remains rather hypothetical [1], [3], [53], [54], and no humerus is preserved with this specimen of “*Proneusticosaurus*.” *Cymatosaurus* skulls were always regarded as morphologically intermediate between pachypleurosaurs and *Nothosaurus*, as are the current “pachypleurosaur” humeri morphotypes I and II. Furthermore, *Cymatosaurus* is a basal member of the Pistosauroidea, including plesiosaurs (Fig. 1A). The occurrence of radial FLB type was already documented in plesiosaurs [25] and very recently also in *Pistosaurus* [26], making it very likely that FLB type occurs through the entire clade and had also developed in *Cymatosaurus*. Thus, size class and morphology of “pachypleurosaur” humerus morphotype I and II, as well as the radial vascularization pattern, and the regular occurrence of well developed layers of fibrolamellar bone in these long bones, support an assignment of histotype A samples to *Cymatosaurus* or a closely related taxon.

For similar reasons, such as size class and abundance of humeri [7], samples showing histotype B are related to the pachypleurosaur *Anarosaurus heterodontus*, although an unequivocal assignment is not possible at the moment.

“Pachypleurosaur” humerus morphotype I and II and histotype A, respectively, now assigned to *Cymatosaurus*, occur in Freyburg and in Winterswijk. In Freyburg, they are the most abundant “pachypleurosaur” humeri but they remain rare in Winterswijk. Histotype B is restricted to Winterswijk. *A. heterodontus* is the only pachypleurosaur known from Winterswijk, and its remains represent the most frequent finds in that locality.

#### Long Bone Histology and Growth Pattern of Histotype A and B Bone Tissue

All sampled Lower Muschelkalk “pachypleurosaurs” have in common a high number of thick and round osteocytes throughout the entire cortex, a basic pattern of radial vascularization, high vascular density, and the regular occurrence of fibrolamellar bone. Differences in histology allow the classification of “pachypleurosaur” long bones into two histotypes. The consistent occurrence of histotype A and B bone tissue, including the three subtypes of histotype B, in humeri and femora, show that the observed histological pattern is reliable and contains a phylogenetic signal. Furthermore, midshaft samples of both histotypes show consistent differences regarding their medullary cavity filling and the deposition of endosteal bone.

Both histotypes show advanced long bone histology. However, histotype A is on the basis of the existing definition [33] assignable neither to the LZB type nor to the FLB type. Histotype B bone tissue type can be summarized as FLB type. Thus, long bone histology of Lower Muschelkalk eosauropterygians reveals an unexpectedly derived bone tissue type, which contrasts with their plesiomorphic humerus morphology. The comparison of the long bone histology of the pachypleurosaur *Anarosaurus* with that of the pachypleurosaur *Neusticosaurus* reveals fundamental differences in bone tissue. On the other hand, Cretaceous plesiosaur long bone histology largely resembles the observations made in *Anarosaurus* (histotype B).

Both “pachypleurosaur” histotypes show different growth patterns. Histotype A always starts with an initial moderate growth phase and continues afterward with alternating phases of fast and slower growth (Fig. 15). Real growth stops in the form of LAGs are rare, but periods of decreased growth are common (annuli). Histotype B shows generally fast growth throughout most of the cortex until growth rate finally decreases in the outer cortex to lamellar zonal bone, or an EFS, respectively, and annuli usually result in LAGs.

The observation that initial growth in histotype A was only moderate or even slow and then increased dramatically is unusual, because in most tetrapods growth starts at high growth rates which then continuously decrease during ontogeny (e.g., [36], [55]). This phenomenon is not understood yet, but could be related to live bearing. Live birth is documented for marine reptiles such as Chinese pachypleurosaurs [13], ichthyosaurs (e.g., [56]), and mosasaurs [57]. Without the large current histological sample, these young individuals could easily have been thought to represent ontogenetic older individuals showing the expected slow growth rate. The occurrence of layers of fibrolamellar bone, mainly in the middle to outer cortex of histotype A, and the alternating changes of fast and slow growth periods, is also unusual, again taking into account that growth rate normally decreases from the inner to the outer cortex as mentioned above.

### Phylogenetic Implications

It was already stated before [40] that bone histology is highly correlated with functional aspects of an individual's life, and is related to external conditions (e.g., climate, habitat) and internal conditions (e.g., life history, growth rates, physiology/metabolic rate). Nevertheless, the same authors also stated that “bone tissue phenotypes can reflect a phylogenetic signal at supraspecific levels if homologous elements are used” [40].

The current study substantiates the hypothesis that bone tissue types contain a clear phylogenetic signal at the level of higher taxa. Details of bone tissue and bone types have never been incorporated in a phylogenetic data matrix to test relationships in a certain group. However, bone histology provides important information along with gross morphology, molecular data, and behavior, and thus, contributes significantly to the understanding of phylogenetic relationships. The only histological data at the moment used for phylogenetic analysis of amniotes were those of the integument, which had increased the overall phylogenetic resolution [58]. Cubo et al. [59], [60] followed a different approach and used statistical methods to quantify histological features and test their value as phylogenetic signal. However, they have not incorporated differences in vascularization (e.g., dominance of radial vascular canals vs. longitudinal vascular canals), bone tissue types (LZB vs. FLB), or growth pattern.

Because phylogenetic hypotheses of Triassic Sauropterygia are unstable (e.g., [9]–[11]), phylogenetic analysis will benefit from additional data. However, the phylogenetic implications for Sauropterygia on the basis of bone histology are discussed here only hypothetically. The real impact of the current histological results on the phylogenetic relationships of Triassic Sauropterygia must be tested in a new phylogenetic analysis.

Current results on long bone histology of Lower Muschelkalk Eosauropterygia, as well as published data on other Sauropterygia [23], [25], [26], have fundamental implications for the relationships within Triassic Sauropterygia. They contradict the accepted phylogeny based on morphology (summarized in [1], Fig. 1A), and support the recently questioned monophyly of pachypleurosaurs ([9], Fig. 1B).

**Figure 17:**
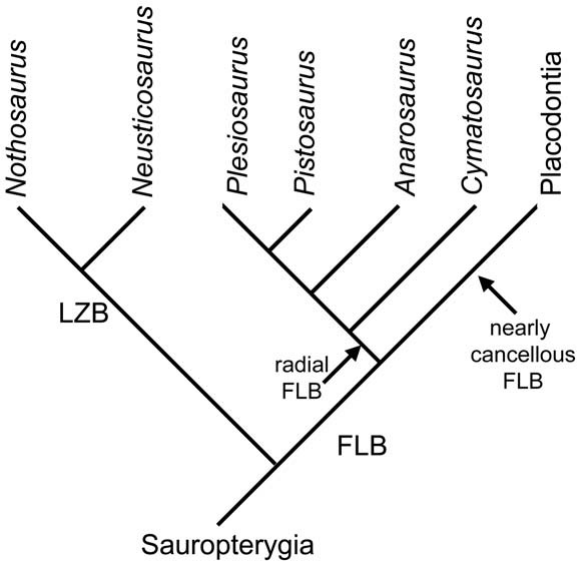
Hypothetical phylogenetic relationships of Sauropterygia based on long bone histology. Patterns of bone tissue types in Sauropterygia long bones contradict the current phylogenetic hypothesis based on morphology as is shown in figure 1. Solely on the basis of the bone histological data, pachypleurosaurs are polyphyletic because *Neusticosaurus* shows lamellar-zonal bone whereas *Anarosaurus heterodontus* has incipient fibrolamellar bone. *Cymatosaurus* already shows a radial vascular pattern and distinct layers of fibrolamellar bone within the parallel-fibered bone tissue. Placodonts have the most advanced bone tissue within Sauropterygia. LZB  =  Lamellar-zonal bone tissue type, FLB  =  Fibrolamellar bone tissue type. Data for pistosaurs and plesiosaurs are taken from the literature [24], [25]. This hypothesis is not yet supported by a data matrix or phylogenetic analysis and thus remains provisional.

Bone histological data hypothetically included in a phylogenetic analysis leads to interesting consequences for the phylogenetic relationships within Sauropterygia: the presence of LZB type in the pachypleurosaur *Neusticosaurus* and in *Nothosaurus*, as well as the presence of FLB type or at least fibrolamellar bone in the pachypleurosaur *Anarosaurus*, in the pistosauroids *Pistosaurus* and *Cymatosaurus*, and in placodonts, suggests that pachypleurosaurs are polyphyletic, and that there are at least three different lineages within Sauropterygia (Fig. 17). One clade is represented by *Nothosaurus* and the pachypleurosaur *Neusticosaurus*, which both share a plesiomorphic growth pattern (LZB type). All other studied Triassic Sauropterygia (Placodontia, Pistosauroidea, and the pachypleurosaur *A. heterodontus*) share an advanced growth pattern (different kinds of FLB type). The pachypleurosaur *Anarosaurus* shows radial FLB type, which is very similar to that observed in pistosaurs [26] and plesiosaurs [25], suggesting a closer phylogenetic relationship than expected on the basis of morphological data. Histotype A, which is assigned to *Cymatosaurus*, the oldest diagnostic sauropterygian and additionally supposed to represent the earliest pistosaur, shows an initial stage of this radial FLB type. However, further studies on the pachypleurosaurs *Dactylosaurus* and *Keichousaurus* are necessary to support the supposed polyphyly of pachypleurosaurs.

Advancement in bone tissue within Triassic sauropterygians is surpassed by placodonts, which show derived humeral morphology along with derived long bone histology, clearly representing a third clade and generally calling into question their basal position in the current phylogenetic hypothesis [1]. Furthermore, the significantly different bone tissues, bone tissue types, and morphology of *Nothosaurus*, *Cymatosaurus*, *Anarosaurus*, and placodonts already occur simultaneously in the Lower Muschelkalk at the very early rise of the Sauropterygia, and did not develop successively through time. The question remains if the fibrolamellar bone in Sauropterygia was inherited from a terrestrial ancestor(s) or represented a new adaptation of the group to the marine life. If fibrolamellar bone was already present in the ancestor of Sauropterygia, this could point to the origin of Sauropterygia within the Archosauria for which fibrolamellar appears to be plesiomorphic [40] rather than within the Lepidosauromorpha which have retained the basal amniote condition of LZB [20], [21], [34]. If fibrolamellar bone represents a new adaptation it could have also evolved multiple times within Sauropterygia.

Bone tissue types may also explain the geographical distribution of Sauropterygia and the extinction of nothosaurs at the end of the Triassic. It is conceivable that FLB type and the accompaniying high metabolic rates allowed the early Pistosauroidea to become globally distributed, because they were able to spread through the colder seas, and finally give rise to the pelagic plesiosaur radiation at the beginning of the Jurassic. The nothosaur clade, which grew with LZB type and presumably lower metabolic rates, depended much more on temperature and external conditions and, thus, remained restricted to the warm epicontinental seas. Finally, nothosaurs became extinct when those warm seas vanished at the end of the Triassic. The extinction of placodonts may be related to their preferred shallow marine habitat and their specialized feeding.

## Materials and Methods

Taxonomic differentiation of eosauropterygian humeri on the basis of standard measurements (e.g., bone length, proximal, shaft, and distal width and length) was tested before but remains difficult [61]. Thus, bone histology is at the moment the best method to study ontogenetic, intra-specific and taxonomical variation in long bones of Eosauropterygia. When possible, bones were sampled exactly at midshaft. In some specimens, sampling locations were proximal or distal to the midshaft on account of preservation (Table S1). All bones were sampled by cutting an entire cross section. Two thin sections are rendered at midshaft from each sample, except for IGWH-22 and IGWH-26/27, which produced four thin sections each along the midshaft area. The sampled bones were afterwards reassembled with plaster (Figs. 3, 4). The samples were cut with a Buehler® Isomet3000 diamond rock saw. The thin sections were ground and polished to a thickness of about 60–80 um using wet SiC grinding powders (SiC 600, 800). Thin sections were then studied with a Leica® DM 2500 compound polarizing microscope, equipped with a digital camera, a Leica® DFC 420C. Different microscopes produced different results because of differences in their optical quality, as pointed out by Castanet et al. [34]. For example, a Leica® DM EP microscope did not show all the growth marks in normal light visible with the Leica® DM 2500.

The morphological descriptions follow the terminology of Rieppel [43] and Bickelmann and Sander [14]. The bone histological terminology follows Francillon-Vieillot et al. [33]. The percentage value of the medullary cavity (Table S1) refers to its cross sectional diameter measured from the preaxial to the postaxial side of the bone. Morphological features like the development of a facet margin as well as the position of the entepicondylar foramen, which were used as ontogenetic features by Sander [12] and Bickelmann and Sander [14], are not considered because they are not applicable to the current sample. If not mentioned otherwise, all humeri show their entepicondylar foramen at a distance from the distal margin and have developed distinct facet margins.

## Supporting Information

Table S1 List of the sampled long bones from the upper Lower Muschelkalk (early middle Anisian) of Freyburg on River Unstrut, Saxony, Germany, and the Lower Muschelkalk (early Anisian) of Winterswijk, The Netherlands.
(0.13 MB DOC)
