# Supplementary material for: Long Bone Histology of Sauropterygia from the Lower Muschelkalk of the Germanic Basin Provides Unexpected Implications for Phylogeny
Source: PLoS One. 2010 Jul 21;5(7):e11613. doi: 10.1371/journal.pone.0011613 (PMC2908119; doi:10.1371/journal.pone.0011613)
Supplement: Table S1 — List of the sampled long bones from the upper Lower Muschelkalk (early middle Anisian) of Freyburg on River Unstrut, Saxony, Germany, and the Lower Muschelkalk (early Anisian) of Winterswijk, The Netherlands. (0.13 MB DOC) [file pone.0011613.s001.doc]

**Table S1. List of the sampled long bones from the upper Lower Muschelkalk (early middle Anisian) of Freyburg on River Unstrut, Saxony, Germany, and the Lower Muschelkalk (early Anisian) of Winterswijk, The Netherlands.**

| **Bone** | **Taxon** | **Repository No.** | **Bl (cm)** | **Mcs** | **Mcc** | **Eb** | **Bt** | **N** |
| --- | --- | --- | --- | --- | --- | --- | --- | --- |
| humerus | *Nothosaurus* sp. | IGWH-3 | 3.6 | 32% | cc, pt | loc. thick | N | 5 |
| humerus | *N. marchicus* | IGWH-25 | 7.65 | 31% | cac, pt | no eb | N | 6 (5) |
| humerus | *N. marchicus* | IGWH-28 | 6.5 | 65% | pm, sm, v | loc. thin | N | 3 (4) |
| humerus | *Nothosaurus* sp. | IGWH-8 | > 7.9 | 75% | cc, pm, sm | loc. thin | N | 5-6 |
| humerus | *Nothosaurus* sp. | IGWH-4 | > 6.55 | 73%* | cc, pm, pt | loc. thin | N | 4 |
| humerus | *Nothosaurus* sp. | IGWH-7 | > 6.25 | 64% | pm, sm, v | no eb | N | 4-5 |
| humerus | *Nothosaurus* sp | IGWH-30 | > 5.75 | X | X | X | X | X |
| humerus | *Nothosaurus* sp. | IGWH-18 | > 5.65 | 65% | cc, cac, pm, pt, sm | loc. thin | N | 5 |
| humerus | *Nothosaurus* sp. | IGWH-14 | > 5.25 | 80% | cc, pm, sm, v | loc. thick | N | 1 |
| humerus | *Nothosaurus* sp. | IGWH-17 | > 4.0 | 75%* | cc, pm, pt | loc. thick | N | 4 |
| humerus | *N. marchicus* | Wijk05-09 | >1.9 | 49%* | cac | loc. thin | N? | 1 (2) |
| humerus | ?*Cymatosaurus* | IGWH-1 | 7.55 | 35% | pm, pt, sm | loc. thick | A | 3 (2) |
| humerus | ?*Cymatosaurus* | IGWH-26/27 | 8.95 | 37% | cac, h, pm, sm | thick | A | 3 (2) |
| humerus | ?*Cymatosaurus* | IGWH-29 | 5.95 | 29% | eb | thick | A | 7 (2) |
| humerus | ?*Cymatosaurus* | IGWH-11 | 4.4 | 51% | cac | thin | A | 0 (1) |
| humerus | ?*Cymatosaurus* | IGWH-19 | > 9.4 | 32% | eb | thick | A | 12-16 (5) |
| humerus | ?*Cymatosaurus* | IGWH-22 | > 5.9 | 19% | eb, h | thick | A | 4 (5) |
| humerus | ?*Cymatosaurus* | IGWH-20 | > 5.3 | 31% | eb | thick | A | 6 (7) |
| humerus | ?*Cymatosaurus* | IGWH-6 | > 5.0 | 21% | cac, sm | loc. thick | A | 4 (5) |
| humerus | ?*Cymatosaurus* | IGWH-31 | > 4.7 | X | X | X | X | X |
| humerus | ?*Cymatosaurus* | IGWH-15 | > 3.9 | * | cc, pt | no eb | A | 2 |
| humerus | ?*Cymatosaurus* | IGWH-10 | > 3.6 | 32% | cc, h, pt | thick | A | 4 |
| humerus | ?*Cymatosaurus* | IGWH-12 | > 3.4 | 75% | cac, pt, sm | thin | A | 0 (1) |
| humerus | ?*Cymatosaurus* | IGWH-16 | > 2.3 | 38% | eb | thick | A | 1 |
| humerus | ?*Cymatosaurus* | NMNHL RGM 449487 | 7.9 | 32% | cac | thin | A | 1 (2) |
| humerus | ?*Cymatosaurus* | NMNHL ST 445912 | 7.2 | X | X | X | X | X |
| humerus | *A. heterodontus* | Wijk08-183 | 5.05 | 27% | cac, h | loc. thick | B | 4 (4) |
| humerus | *A. heterodontus* | Wijk09-58 | 4.9 | 32% | cac, sm | loc. thick | B | 6 (3) |
| humerus | *A. heterodontus* | Wijk09-472 | 4.35 | 36% | cac | thin | B | 0 (2) |
| humerus | *A. heterodontus* | Wijk07-50 | 4.15 | 39% | cac | thick | B | 3 (4) |
| humerus | *A. heterodontus* | Wijk09-543 | 3.6 | 26% | cac | thick | B | 2 (2) |
| humerus | *A. heterodontus* | Wijk08-219 | 3.4 | 32% | h | thick | B | 1 (2) |
| humerus | *A. heterodontus* | Wijk07-137 | ~ 3.0 | 50% | cac, sm | loc. thick | B | 4 |
| humerus | *A. heterodontus* | Wijk07-70 | > 3.6 | 29% | cac, sm | loc. thick | B | 2 (2) |
| humerus | *A. heterodontus* | Wijk06-238 | >2.0 | 50% | pm, pt, sm | loc. thick | B | 0 (1) |
| ?humerus | *A. heterodontus* | NME48000085c | frg. | 40% | cac, sm | no eb | B | 0 (1) |
| humerus | placodont | IGWH-9 | 6.8 | 33% | pt | no eb | P | 3 (4) |
| femur | placodont | IGWH-23 | > 5.7 | 60% | pt | no eb | P | 5 (3) |
| femur | *Nothosaurus* sp. | Wijk05-10 | 9.3 | 60% | cac, pt | no eb | N | 8 |
| femur | ?*Cymatosaurus* | IGWH-21 | >7.7 | 27% | h, sm | thick | A | 12 (4) |
| femur | ?*Cymatosaurus* | IGWH-24 | > 4.9 | 34% | pt, sm | thin | A | 7 (2) |
| femur | ?*Cymatosaurus* | IGWH-2 | 4.7 | 55% | cc, pm, sm | no eb | A | 1 (2) |
| femur | ?*Cymatosaurus* | IGWH-5 | > 2.9 | 27% | pt, sm | loc. thick | A | 2 (2) |
| femur | *A. heterodontus* | NME48000075 | frg. | 28% | cac, sm | thick | B | 2 (2) |
| femur | *A. heterodontus* | Wijk08-150 | 6.8 | 17% | cac, sm | loc. thick | B | 3 (5) |
| femur | *A. heterodontus* | Wijk07-03 | 6.2 | 50% | pm, sm | thin | B | 2 (3) |
| femur | *A. heterodontus* | Wijk06-102 | > 3.5 | 19% | cac | loc. thick | B | 6 |
| femur | *A. heterodontus* | Wijk06-14 | > 3.4 | 14% | X | ? loc. thick | B | 3 (2) |
| femur | *A. heterodontus* | Wijk06-86 | > 2.3 | 34% | sm | loc. thin | B | 2 |
| femur | *A. heterodontus* | Wijk07-11 | > 4.05 | 26% | cac | no eb | B | 0 (1) |
| femur | *A. heterodontus* | WijkHO-A568 | > 2.0 | 24% | cac | no eb | B | 1 (2) |
| femur | *A. heterodontus* | Wijk06-84 | 4.05 | 25% | cac | loc. thin | B | 1 (2) |
| femur | ?*Cymatosaurus* | NME48000085a | frg. | 35% | cac,  sm, pt | thin | A | 4 (2) |
| femur | ?*Cymatosaurus* | NME48000074 | frg. | 22%* | eb, h | thick | A | 7 (3) |
| femur | *A. heterodontus* | Wijk09-636 | frg. | 35% | cac | loc. thin | B | 0 (3) |
| humerus | ? pachypleurosaur | IGWH-13 | > 5.45 | 15% | eb | thick | X | 9 (3) |

Listed bones are inventoried in the IGWH, NMNHL (including “Wijk”), and NME. Abbrevations in alphabetical order. Bt = bone tissue. [N represents the typical bone tissue of nothosaurs, A and B represents the bone tissues as observed in the current “pachypleurosaur” sample (histotype A and B), P represents the bone tissue of placodonts, and X represents bone tissue unknown or unassigned.]; Bl = bone length [Frg. = bone fragment, > = incomplete bone]; Mcs = size of the medullary cavity measured at midshaft in pre-postaxial direction of the cross sectional diameter [* = sampling location not at midshaft]; Mcc = content of medullary cavity [cac = calcite crystals, cc = calcified cartilage, eb = mainly filled by endosteal bone, h = hollow, pm = primary matrix, pt = primary trabeculae, sm = secondary matrix, v = vesicles]; [loc = locally]; N = number of counted growth marks, with the number of alternating growth phases in parentheses (see text for further explanation). The taxonomic assignment of femora is based solely on their histology.
